# Effective suppression of Dengue virus using a novel group-I intron that induces apoptotic cell death upon infection through conditional expression of the Bax C-terminal domain

**DOI:** 10.1186/1743-422X-11-111

**Published:** 2014-06-13

**Authors:** James R Carter, James H Keith, Tresa S Fraser, James L Dawson, Cheryl A Kucharski, Kate M Horne, Stephen Higgs, Malcolm J Fraser

**Affiliations:** 1 Department of Biological Sciences, Eck Institute of Global Health, University of Notre Dame, Notre Dame, Indiana 46556, USA; 2 Biosecurity Research Institute, Kansas State University, Manhattan, Kansas 66506, USA

**Keywords:** Dengue, *trans*-splicing, Group I intron, Ribozyme, Mosquito, Antiviral, Suppression

## Abstract

**Introduction:**

Approximately 100 million confirmed infections and 20,000 deaths are caused by Dengue virus (DENV) outbreaks annually. Global warming and rapid dispersal have resulted in DENV epidemics in formally non-endemic regions. Currently no consistently effective preventive measures for DENV exist, prompting development of transgenic and paratransgenic vector control approaches. Production of transgenic mosquitoes refractory for virus infection and/or transmission is contingent upon defining antiviral genes that have low probability for allowing escape mutations, and are equally effective against multiple serotypes. Previously we demonstrated the effectiveness of an anti-viral group I intron targeting U143 of the DENV genome in mediating trans-splicing and expression of a marker gene with the capsid coding domain. In this report we examine the effectiveness of coupling expression of ΔN Bax to trans-splicing U143 intron activity as a means of suppressing DENV infection of mosquito cells.

**Results:**

Targeting the conserved DENV circularization sequence (CS) by U143 intron trans-splicing activity appends a 3’ exon RNA encoding ΔN Bax to the capsid coding region of the genomic RNA, resulting in a chimeric protein that induces premature cell death upon infection. TCID50-IFA analyses demonstrate an enhancement of DENV suppression for all DENV serotypes tested over the identical group I intron coupled with the non-apoptotic inducing firefly luciferase as the 3’ exon. These cumulative results confirm the increased effectiveness of this αDENV-U143-ΔN Bax group I intron as a sequence specific antiviral that should be useful for suppression of DENV in transgenic mosquitoes. Annexin V staining, caspase 3 assays, and DNA ladder observations confirm DCA-ΔN Bax fusion protein expression induces apoptotic cell death.

**Conclusion:**

This report confirms the relative effectiveness of an anti-DENV group I intron coupled to an apoptosis-inducing ΔN Bax 3’ exon that trans-splices conserved sequences of the 5’ CS region of all DENV serotypes and induces apoptotic cell death upon infection. Our results confirm coupling the targeted ribozyme capabilities of the group I intron with the generation of an apoptosis-inducing transcript increases the effectiveness of infection suppression, improving the prospects of this unique approach as a means of inducing transgenic refractoriness in mosquitoes for all serotypes of this important disease.

## Introduction

Dengue virus (family Flaviviridae) is maintained in a cycle between humans and the widely distributed *Aedes aegypti* mosquitoes [1,2]. Approximately 100 million infections and 20,000 deaths each year are attributed to mosquito-borne DENV infections. An additional 2.5 billion people worldwide remain at risk making this virus one of the most critically important pathogens in the world [3]. However, a more severe global scenario of dengue virus infections has been presented. Bhatt et al. estimated using cartographic models that there are as much as 390 million dengue virus infections annually [4]. The reason for the discrepancy is that approximately 290 million are asymptomatic or mild ambulatory infections that have no need for clinical management and are unrecorded. Asymptomatic infections affect precise determination of economic impact, elucidation of population dynamics of dengue viruses [4], and establishment of future vaccination programs.

The WHO has reported outbreaks in Key West, Florida in 2009 and 2010, Puerto Rico in 2010, and Miami-Dade County and Pakistan in 2011 [5-8]. Most recently, the WHO reported dengue outbreaks in 2012 on the Madeira Islands of Portugal [9,10] resulting in over 2000 cases, with imported cases detected in 10 other countries in Europe [9,10]. In 2013, dengue cases were detected in Florida (United States of America) [9]. Dengue continues to be a major burden in several South American countries, most notably Brazil, Honduras, Costa Rica and Mexico [9,11-14]. In Asia, an increase in cases were reported in Singapore after a lapse of several years, and outbreaks have been reported in Laos, and the Chinese province of Yunnan [9]. In 2014, after an absence of over 10 years data indicates increases in the number of dengue cases in the Pacific countries of the Cook Islands, Malaysia, Fiji and Vanuatu [9].

Infection with one of four orthologous, but antigenically distinct DENV serotypes (designated DENV 1 through 4) can result in dengue fever (DF) or dengue hemorrhagic fever (DHF) [3]. DF and DHF are endemic to tropical and subtropical regions of the world, but global changes in climate, rapid dispersal of virus due to travel and commerce, and increased human migration to non-tropical regions has resulted in epidemic DENV outbreaks in areas that are non-endemic for these viruses [1,2]. There are currently no consistently effective preventive control measures or approved tetravalent vaccines to combat DENV.

Population replacement of vector competent mosquitoes with transgenic mosquitoes that are refractory for virus infection and/or transmission has been proposed as a potentially long lasting, cost effective, and safe control measure for interrupting the dengue disease transmission cycle [15,16]. The success of this approach relies, in large part, upon defining DENV suppressive approaches that limit or prevent the evolution of escape mutants, and are effective against multiple strains and serotypes.

Our lab has been surveying ribozymes as DENV suppressive agents for use in generating refractory transgenic mosquitoes. In a previous report we examined the effectiveness of hammerhead ribozymes in suppressing DENV infection in retrovirus transduced mosquito cells [17]. We identified several hammerhead ribozymes that were effective in significantly reducing DENV serotype 2 New Guinea strain (DENV2-NGC) infection of *Ae. albopictus* C6/36 cells. However, the inability to target sequences that are conserved among all DENV serotypes by this method necessitated exploration of ribozymes that have an increased potential for broader specificity. As an alternative we demonstrated the utility of a group I intron *trans*-splicing strategy to target circularization sequences (CS) that are highly conserved among all DENV genomes [18].

*Trans*-splicing group I introns provide a versatile tool for repairing erroneous or unwanted RNA [19-29], and have been used for a number of applications, including repair of defective α-globin mRNA [20], renovation of wild-type p53 function [30], re-establishment of canine skeletal muscle chloride channel function [31], and induction of p16 activity in a pancreatic cell line [22]. More applicable to our research are examples of *trans*-splicing group I introns targeting the HIV-1 tat sequence [32], cucumber mosaic virus coat protein mRNAs [19], and the hepatitis C virus internal ribosome entry site (HCV-IRES; [33]).

Any method of inhibition of DENV infection by interaction with the RNA genome must be designed to target highly conserved sequences [34-36]. The 5’ and the two 3’ cyclization sequences (5’ CS, CS1, and CS2) of the DENV genome are the most invariant segments, and are essential for formation of the panhandle structure required for genome replication [37,38]. The 5’ CS is located downstream of the polyprotein start codon, well within the ORF of the Capsid (CA) protein sequence. The stringency of tolerable mutations in this sequence among DENV may be increased, in part, by the need for the virus to conserve a functional CA protein. Moreover, all mosquito-borne flaviviruses share an 8bp stretch of nucleotides within this 5’ CS sequence [39].

We previously demonstrated the effectiveness of group I introns targeting sequences in the DENV 5’ CS [18]. While these group I introns demonstrated the capability to splice within this conserved region, the use of these molecules as simple catalytic genome destroying agents necessarily requires levels of expression that match or exceed those for the generation of viral genomes in infected cells. Escape mutants may emerge in the event virus replication exceeds group I intron catalytic suppression. As a result, expression of anti-viral group I introns alone in cells may not be the ideal method for decreasing the probability of generating escape mutants while suppressing DENV expression. Coupling the splicing activity of the group I intron to a death-upon-infection strategy could provide an added level of insurance against the emergence of escape mutants. Designing anti-DENV group I introns coupled with apoptosis-inducing genes as the 3’ exons to induce cell death upon infection with DENV would increase the effectiveness of infection suppression and could diminish the probability of escape mutant emergence.

This report confirms the relative effectiveness of an anti-DENV group I intron coupled to an apoptosis-inducing ΔN Bax 3’ exon that *trans*-splices conserved sequences of the 5’ CS region of all DENV serotypes and induces apoptotic cell death upon infection. The proapoptotic ΔN Bax was chosen as the 3’ exon because of its ability to irreversibly initiate apoptosis more rapidly than Bax [40] due to deletion of the Bax BH-3 domain that facilitates protein-protein interactions between Bax and Bcl-2 or other anti-apoptotic regulators. We demonstrate this introns’ antiviral and apoptosis-inducing activity in transformed mosquito cell cultures challenged with infectious DENV of all four serotypes. Our results confirm that coupling the targeted ribozyme capabilities of the group I intron with the generation of an apoptosis-inducing transcript increases the effectiveness of infection suppression and improves the prospects of this unique approach as a means of inducing transgenic refractoriness in mosquitoes for all serotypes of this important disease.

## Results

### Basic *trans*-splicing mechanism of group I introns

**Figure 1:**
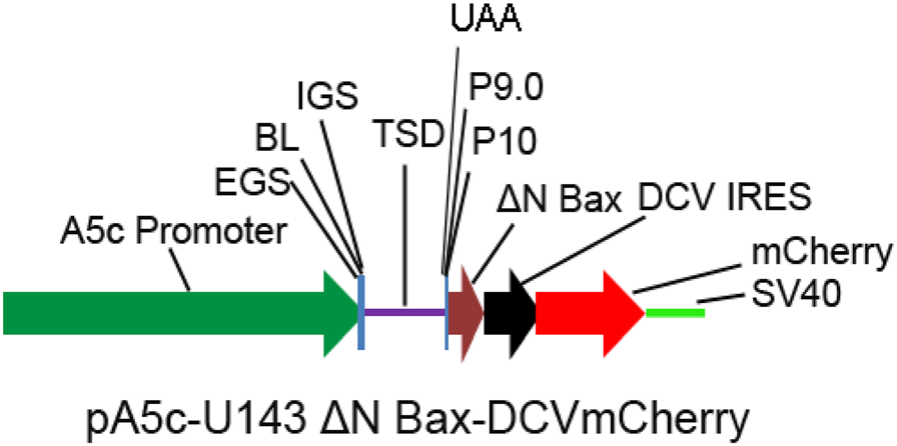
**Anti**-**DENV group I intron targeting and *trans***-**splicing DENV targets.** Illustration of the anti-DENV group I intron, αDENV-U143, targeting the DENV genome and the associated *trans*-splicing reaction. The targeted 5’ CS domain of the DENV genome is conserved among all serotypes [18]. The uracil at position 143, located within the 5’ CS domain, is targeted for cleavage. Ribozyme and target DENV genomic (blue) RNAs are indicated. IGS = Internal guide sequence, EGS = external guide sequence, CS = cyclization sequence.

The *trans*-splicing mechanism of the *Tetrahymena thermophila* group I intron is characterized by two independent transesterification reactions ( [41]; Figure 1). Ribozyme and target RNAs base pair to form the P1 and extended antisense helices with the subsequent guanosine-mediated transesterification resulting in cleavage of the target DENV RNA (Step 1). The external guide sequence (EGS) provides additional stability for the *trans*-splicing reaction through Watson-Crick base pairing with nucleotides downstream from the reactive uracil on the target RNA. The distal portion of helix P1 is displaced by sequences from the 3’ exon (ΔN Bax) to form helix P10 (Step 2). This allows the second transesterification to proceed, resulting in ligation of the DENV capsid and ΔN Bax (Step 3). The end result is a new RNA molecule that, if appropriately configured, can be translated into a fusion protein sequence.

### Construction of the αDENV-U143-ΔN Bax vectors

In a previous report we describe the construction and *trans*-splicing activity of a U143 intron that effectively *trans*-splices all known DENV genomes by targeting the uracil at position 143 within the 5’ CS [18]. This *trans*-splicing anti-DENV group I intron, designated αDENV-U143, includes a 9 nucleotide EGS that base pairs to sequences conserved among all DENV genomes to improve the targeting capability of the intron and to minimize potential off-target splicing interactions. An internal guide sequence (IGS) of 9 bases includes a reactive guanosine that forms a wobble base pair with the targeted uracil. A single variable base at nucleotide 152 is positioned within a non-homologous bulge loop (BL) structure that separates the IGS and EGS, and therefore does not influence the targeting of the intron [18]. This BL structure allows the formation of the P10 helix which increases the catalytic efficiency of the intron [42]. Excluding the wobble base with the uracil at nucleotide position 143 which is required for proper cleavage [43-46], 17 bases of this intron interact directly with the intended target sequence.

**Figure 2:**
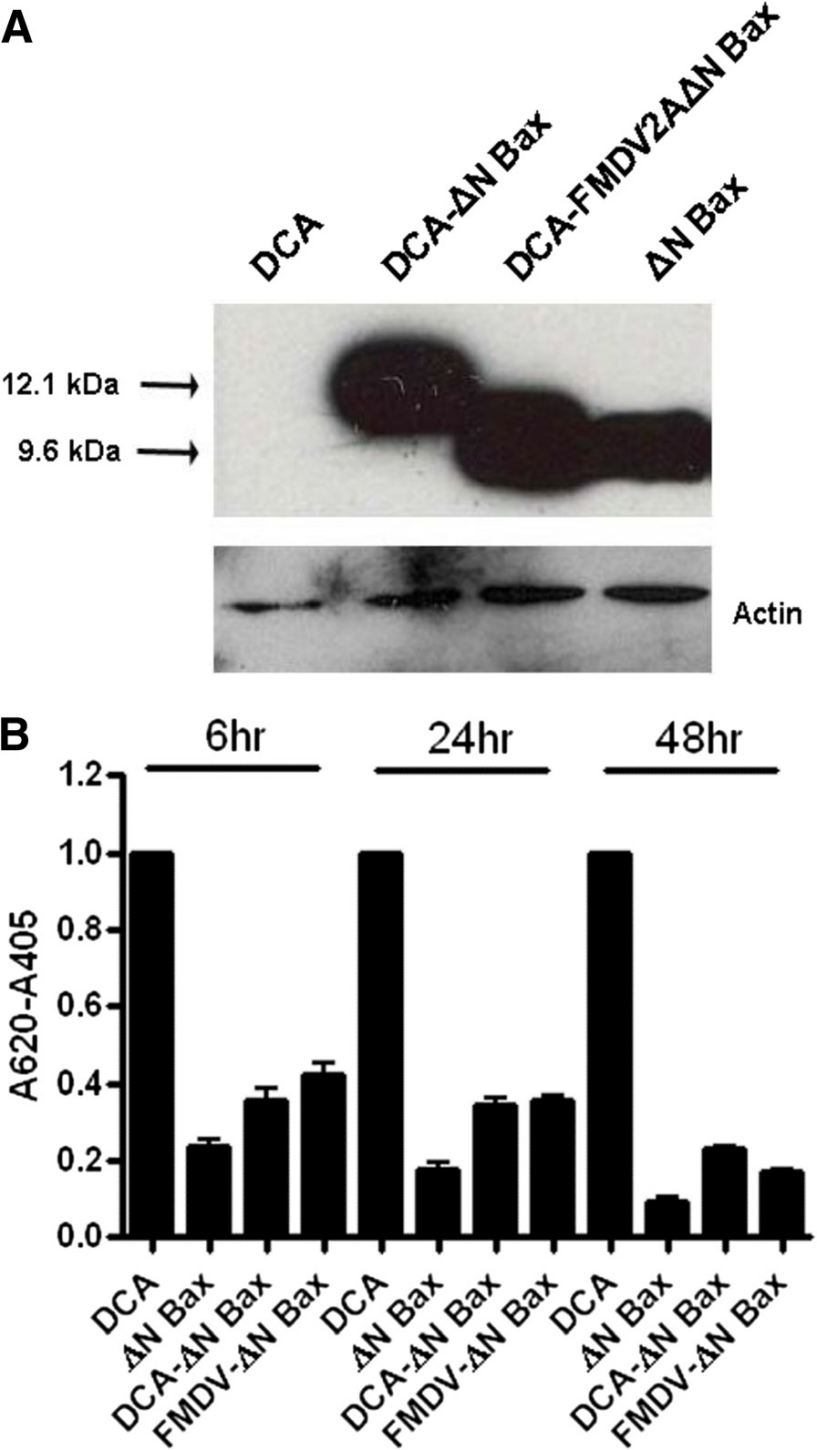
**Schematic of the group I (*trans***-**splicing) intron expression cassette.** Each *trans*-splicing αDENV-U143 group I intron was tagged downstream of the ΔN Bax 3’ exon with the mCherry fluorescent marker gene expressed from the Drosophila C virus (DCV) IRES sequence. Expression of this construct was driven by the *Drosophila melanogaster* Actin 5c promoter (A5c). This bi-cistronic configuration allowed monitoring for the presence and expression of the anti-DENV group I intron constructs within cell cultures. A5c = *Drosophila* Actin 5c promoter, EGS = External guide sequence, IGS = Internal guide sequence, TSD = *Trans*-splicing domain, ΔN Bax = pro-apoptotic 3’ exon, IRES = Internal ribosome entry site.

In this study we modified αDENV-U143 by incorporating a ΔN Bax coding sequence as the 3′ exon (Figure 2). The ΔN Bax sequence corresponds to amino acids 112–192 of the Bax protein sequence, and induces cell death in A549 and NCI-H1299 cell lines more efficiently than tBax through a caspase-independent mechanism [40]. To insure that this potent apoptosis inducer was not expressed independently of splicing from the αDENV-U143-ΔN Bax intron we inserted a UAA stop codon into the P9.0 helix of the group I intron immediately upstream of the UCG splice donor (Figure 2).

We also constructed an inactive negative control group I intron, αDENV-ΔU143, by removing the entire catalytic core, domains P4 through P6, of αDENV-U143 [18] as described in Methods. The removal of domains P4 through P6 was necessary since a previously described ΔP5abc intron had demonstrated residual *trans*-splicing activity [47]. We further modified the αDENV-U143-ΔN Bax by attaching a Drosophila C Virus (DCV) IRES-dependent mCherry fluorescent marker gene downstream of the ΔN Bax 3’ exon. The DCV dicistrovirus IRES sequence previously yielded high levels of expression *Ae. aegypti* mosquito cells [48]. This bi-cistronic configuration allowed monitoring for the presence and expression of the anti-DENV group I intron constructs within mosquito cell cultures. The entire construct was designed to be expressed in mosquito cells using the *Drosophila melanogaster* Actin 5c promoter (A5c).

### Expression of ΔN Bax initiates apoptosis

**Figure 3:**
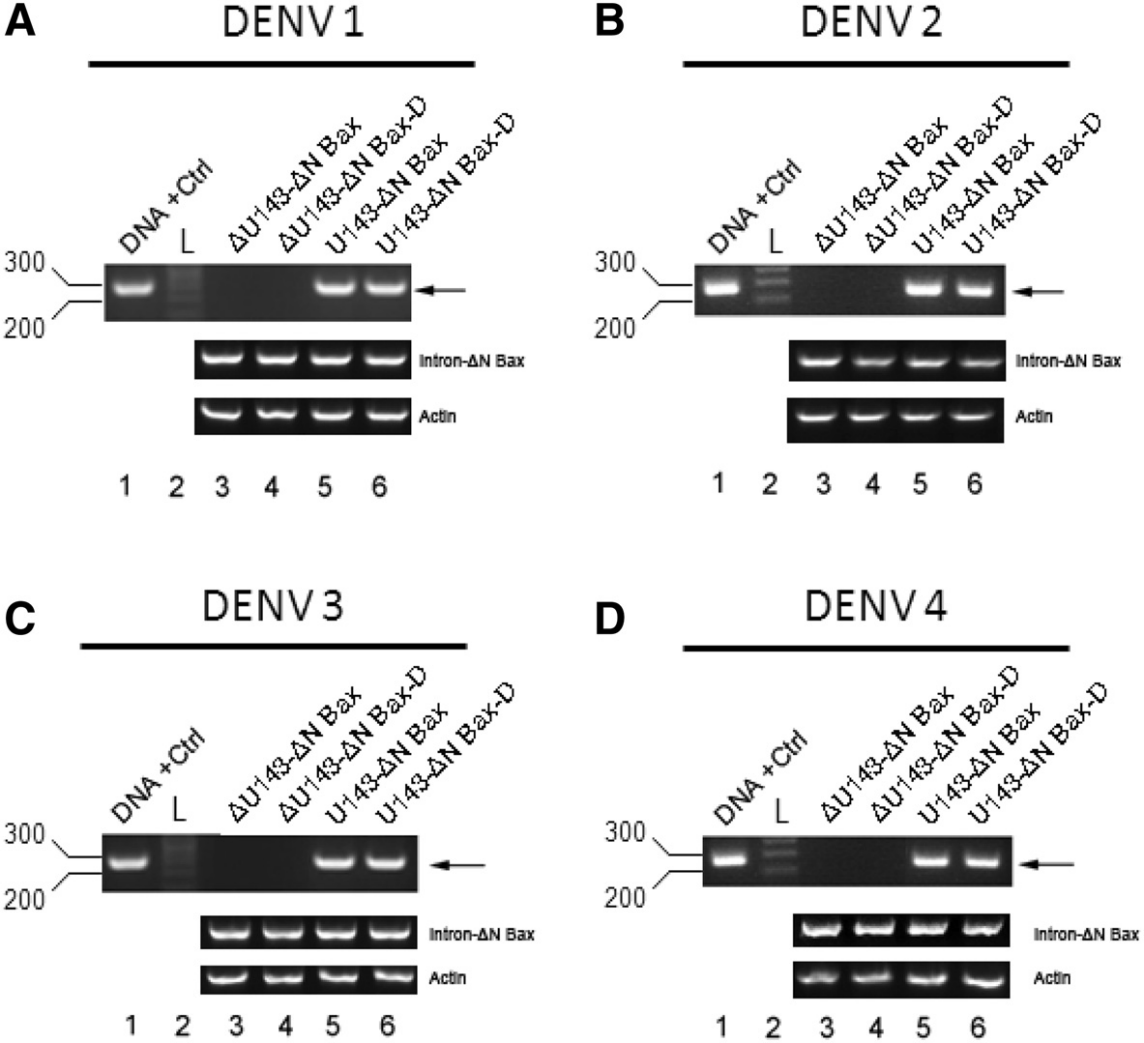
**Assessment of ΔN Bax expression and proapoptotic activity. A**. Western blots were performed as described in Methods. DCA, DCA-ΔN Bax, FMDV2A-ΔN Bax, and ΔN Bax probed with Bax C-terminal specific antibody. The bottom bands are corresponding actin loading controls. **B**. The relative amount of amido black recovered from each well is inversely proportional to the extent of apoptosis. The DCA wells were set to a value of 1 for each time point and the readings for all other wells at each time point were adjusted by the corresponding ratio [50]. Measurements were recorded in triplicate for each time point. Statistical analysis was performed using ANOVA test with Tukey post test. Differences were statistically significant with p values less than 0.05.

The αDENV-U143-ΔN Bax group I intron cleaves within 19 amino acids of the terminus of the DENV polyprotein open reading frame at U143 and appends the ΔN Bax 3’ intron to the DENV CA protein coding sequence (DCA). We analyzed the relative effectiveness of expression of this DCA-ΔN Bax fusion protein from this predicted RNA configuration, and verified its capabilities as an inducer of apoptosis in C6/36 cells. The copper sulfate-inducible metallothienine promoter (pMT) was employed to control expression of transcripts encoding the apoptosis negative control DCA, the predicted DCA-ΔN Bax fusion protein, the unaltered ΔN Bax protein, or a DCA-FMDV2A-ΔN Bax transcript that was predicted to independently express ΔN Bax from DCA. At 48 hours post-induction proteins were harvested and analyzed by western blot with anti-Bax antibody as described in Methods. No protein bands were evident from the DCA lysates (Figure 3A) while the DCA-ΔN Bax fusion protein (12.1 kb) and ΔN Bax (9.6 kb) were produced at similar levels from their respective expression constructs, demonstrating that the 19 amino acid DCA extension of the N-terminus of ΔN Bax has no observable effect on the efficiency of expression of the protein product.

Physiological determinants of apoptosis such as cellular shrinkage and detachment [49] may be conveniently assayed by amido black staining [50,51]. To confirm retention of apoptotic activity for the DCA-ΔN Bax fusion, we performed amido black assays on C6/36 cells co-transfected with the pMT-EYFP induction marker plasmid and each of the inducible DCA, ΔN Bax, DCA-ΔN Bax, and DCA-FMDV2A-ΔN Bax expression plasmids (Figure 3B). Cells expressing the DCA negative control displayed a significant difference in cell detachment at each time point compared to cells expressing some form of ΔN Bax. No significant differences were observed among the values obtained for the pMT-ΔN Bax or pMT-DCA-ΔN Bax transfected cells at any time point. While this assay was not considered definitive for quantitating apoptosis activity, the results were consistent with the induction of an apoptotic response by the DCA-ΔN Bax fusion protein.

### The αDENV-U143-ΔN Bax intron construct effectively targets all DENV serotypes

**Figure 4:**
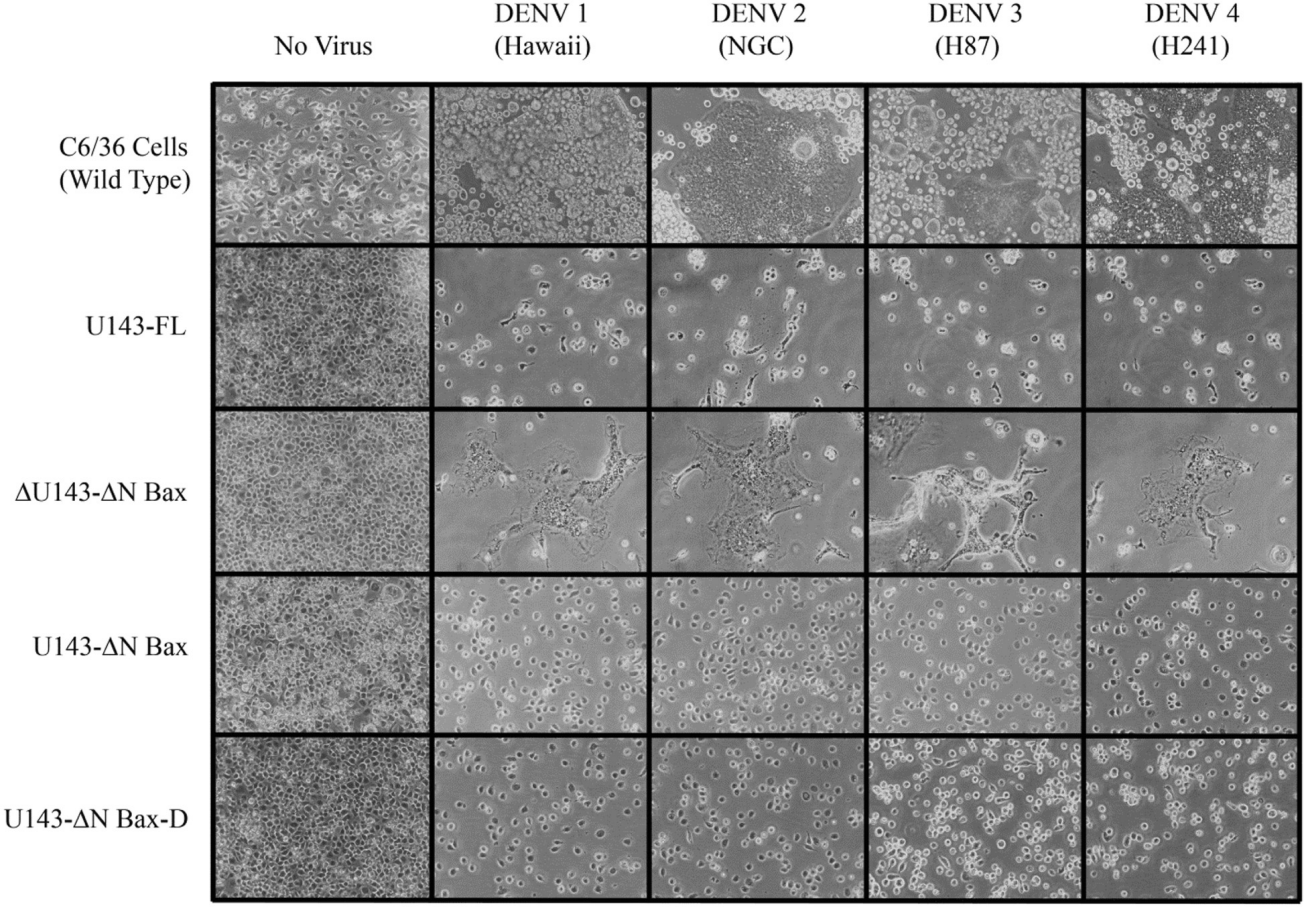
**αDENV**-**U143**–**ΔN Bax constructs effectively target all DENV serotypes A). ***Ae. albopictus* C6/36 cells were transformed with *trans*-splicing (αDENV-U143) or inactive (αDENV-ΔU143) group I intron vector constructs and maintained under hygromycin selection. U143-ΔN Bax refers to the αDENV-U143 *trans*-splicing group I intron possessing the pro-apoptotic ΔN Bax as the 3’ exon. ΔU143-ΔN Bax refers to the anti-DENV group I intron (αDENV-ΔU143) possessing the inactive deletion mutation of the *trans*-splicing domain that is linked to the ΔN Bax 3’ exon. The deletion mutation of the *trans*-splicing domain is designed to knock out *trans*-splicing function, providing a negative control [81]. At 15 hours post plating 5x10^6^ cells were infected with **A**. DENV-1, **B**. DENV-2, **C**. DENV-3, **D**. DENV-4, each at MOI 0.1, and analyzed for the presence of splice product 96 hours p.i. by RT-PCR with heterologous primers. A PCR amplification product derived from a separately constructed spliced sequence control (DNA + Ctrl, see Methods) and a DNA ladder (L) are provided as size standards for each gel. U143-ΔN Bax-D and ΔU143-ΔN Bax-D refer to the active and inactive intron-ΔN Bax constructs respectively that are linked to the DCV –IRES/mCherry configuration as shown in Figure 2. Control RT-PCR experiments were performed with primers for actin to confirm similar RNA loading. Heterolgous primers to the intron- ΔN Bax segment of the construct were used to confirm the presence of our anti-DENV introns. Arrows indicate the predicted size of the principle splice products resulting from intron activity. The identity of spliced products was confirmed by sequencing.

We examined the effectiveness of αDENV-U143-ΔN Bax to target all DENV serotypes in transformed *Ae. albopictus* C6/36 cells (Figure. 4A through D). Intron-expressing cell lines were generated by co-transfection of C6/36 cells with a hygromycin selectable marker plasmid and A5c promoter plasmids expressing either the inactive αDENV-ΔU143-ΔN Bax or the *trans*-splicing αDENV-U143-ΔN Bax introns that were each linked and unlinked to a DCV-IRES/ mCherry. Following selection, the transformed C6/36 cells were seeded at a density of 2.0 × 10^5^ cells/cm^2^ per T25 flask, and were challenged with each of the four DENV serotypes at 0.1 MOI.

Total cellular RNA isolated at 96 hours post-infection was assessed for αDENV-U143-ΔN Bax activity by RT-PCR amplification of *trans*-spliced products and the identity of these product bands were confirmed by sequencing. Spliced products of approximately 250 bp with appropriate junction sequences were observed as a result of targeting the DENV genome, regardless of serotype (Figure 4). Targeting and cleavage occurred whether the αDENV-U143-ΔN Bax configuration was linked or unlinked to a DCV-IRES/mCherry extension. As expected the αDENV-ΔU143 inactive anti-DENV group I intron did not catalyze a *trans*-splicing reaction.

### Expression of αDENV-U143-ΔN Bax introns from the A5c promoter appends 56 nt of RNA sequence to the 5’ terminus

Previous analysis of αDENV group I introns using the A5c promoter [18] demonstrated targeting and cleavage effectiveness against both synthetic targets and viral genomes. Of concern was the possibility that the catalytic activity might be influenced by variability in the length of the 5’ terminus of the expressed transcript, effectively causing varying degrees of interference with the proper alignment between the external or internal guide sequences and the target genome.

The putative transcription start site (TSS) of the A5c promoter was previously reported as being at 56 nucleotides upstream of the 5’terminus of our αDENV-U143-ΔN Bax group I intron sequence (Additional file 1: Figure S1A; [52]). We determined the precise TSS of our anti-DENV group I intron transcripts using 5’-RLM-RACE analysis (RNA Ligase Mediated-Rapid Amplification of cDNA Ends) on the expressed αDENV-U143-ΔN Bax intron. Since all expression plasmids possessed the same promoter and targeting sequences (i.e. EGS and IGS) there was no need to perform this assay on all constructs. Gel electrophoresis of RT-PCR products resulted in a distinct single product band of approximately 250 bases (Additional file 1: Figure S1B), as expected for a 56 nt 5’ extension, indicating a homogeneous transcript length and TSS (Additional file 1: Figure S1C).

### Comparative CPE assays

**Figure 5:**
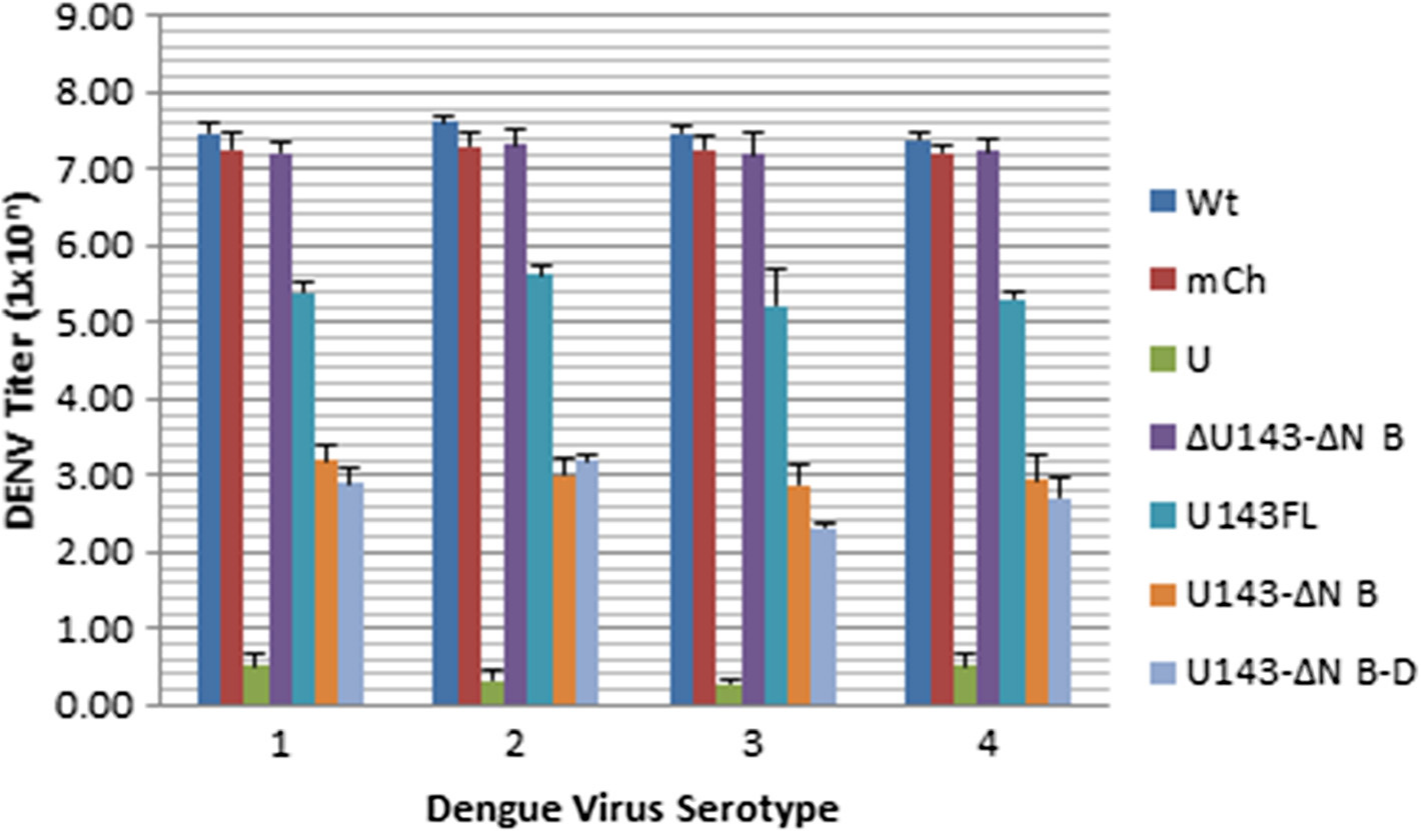
**CPE following DENV infection of group I intron transformed C6/36 cells.** C6/36 cells were transformed with the anti-DENV group I introns or mCherry expression plasmids, and challenged with DENV (MOI 0.1). Micrographs were taken at 6 dpi using an inverted phase microscope at 20× magnification. Representative infected cell cultures are shown. Cultures expressing functional αDENV-U143-ΔN Bax intron constructs exhibited no CPE while some degree of cytopathology was observed for αDENV -U143-FL and the negative control αDENV -ΔU143-ΔN Bax. αDENV-U143-ΔN Bax-D refers to the active intron-ΔN Bax construct that is linked to the DCV-IRES/mCherry configuration as shown in Figure 2.

DENV infection of C6/36 cells causes decreased cell proliferation and a distinctive syncytium-inducing cytopathic effect (CPE; [53,54]) mediated by cell surface expression of the DENV envelope protein (DENV-E). If expression of the αDENV-U143-ΔN Bax construct renders mosquito cells refractory to DENV replication, absence of substantial CPE should be evident.CPE was most noticeable upon DENV infection of C6/36 cells in the absence of the active U143-ΔN Bax, or in the presence of the inactive anti-DENV group I intron, αDENV-ΔU143-ΔN Bax (Figure 5). Infection of C6/36 cells expressing the αDENV-U143-firefly luciferase (FL) group I intron induced significantly less CPE than either untransformed or αDENV-ΔU143-ΔN Bax transformed cells, demonstrating that expression of an active group I intron is able to suppress DENV infection regardless of the 3’ exon used. In contrast, cells expressing the αDENV-U143-ΔN Bax construct, whether linked with a DCV-IRES/mCherry configuration or not, had no detectable CPE following DENV infection. Incorporation of the 3’ ΔN Bax exon controlled infection of the cell cultures better than the expressed αDENV-U143 group I intron alone, and suggested an improved effectiveness with the apoptosis induction.

As expected, CPE was not evident in the absence of DENV infection, but was observed following infection of control C6/36 cells transformed with an A5c-promoted mCherry plasmid. The latter control established that absence of CPE following DENV infection of αDENV-U143-FL or αDENV-U143-ΔN Bax intron transformed cells was not attributable to hygromycin-mediated inhibition of DENV replication.

### Quantitative assessment of the relative effectiveness of ΔN Bax as the 3’ exon

**Figure 6:**
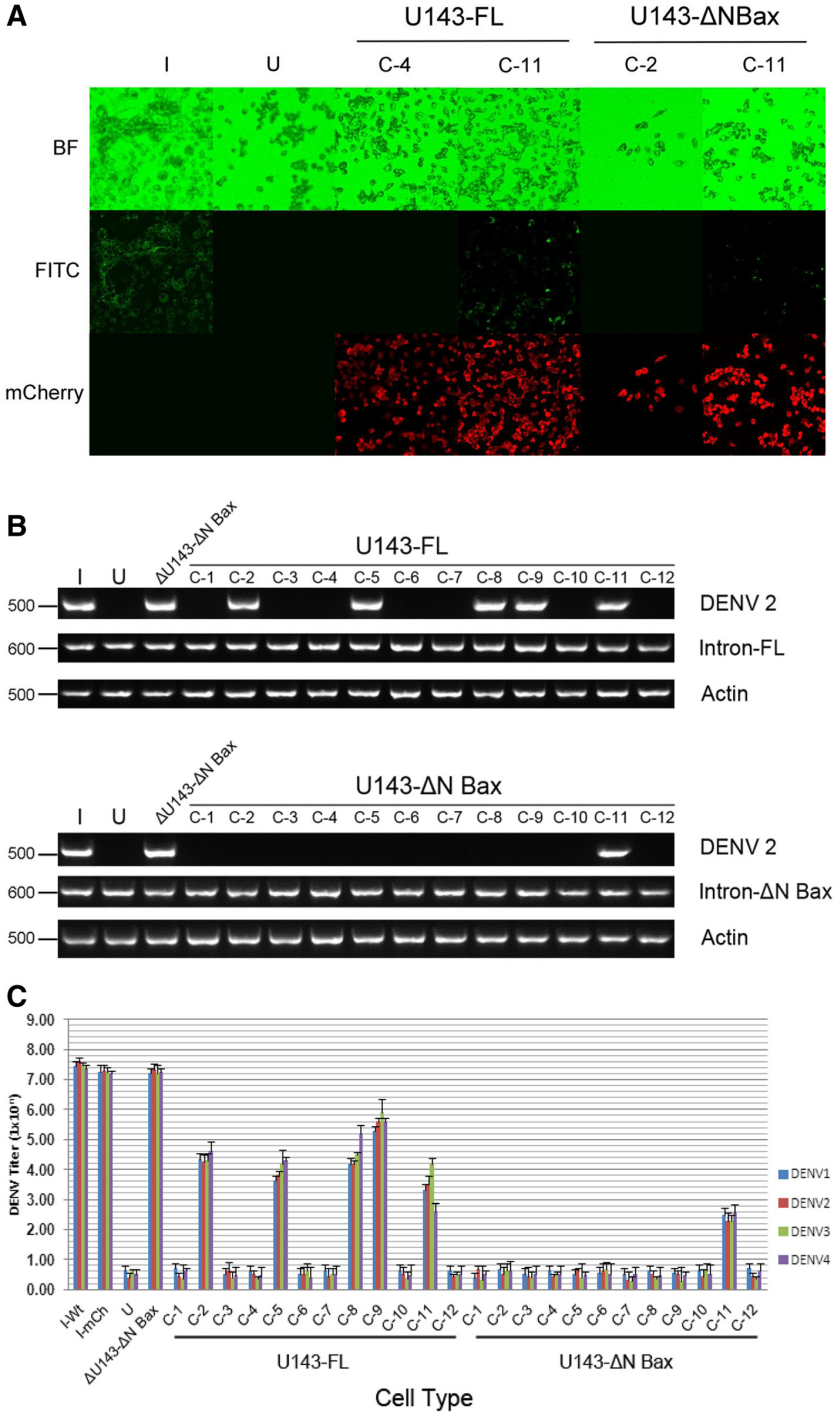
**U143**–**ΔN Bax constructs effectively suppresses DENV in transformed mosquito cells.** C6/36 cells transformed with anti-DENV group I introns were challenged with the DENV serotypes 1 through 4 (MOI 0.01). At 4 dpi supernatants were collected and viral titers were determined by TCID_50_-IFA as described in [17]. Suppression of DENV replication is determined through detection of the cell surface expressed DENV E protein. Only in mosquito cells expressing a functional αDENV-U143-ΔN Bax construct were suppression of DENV evident. Suppression of viral replication occurred with all serotypes tested, and was increased when FL was replaced by αDENV-U143-ΔN Bax or αDENV-U143-ΔN Bax-D as the 3-exon. U143-ΔN Bax-D refers to the active αDENV-U143-ΔN Bax construct that is linked to the *Drosophila* C virus DCV IRES/mCherry configuration as shown in Figure 2. I-Wt = infected naïve C6/36 cells. I-mCh = DENV infected C6/36 cells transformed with a construct constitutively expressing the mCherry fluorescent marker. U = uninfected naïve C6/36 cells. ΔN B = ΔN Bax.

By observing suppression of CPE in C6/36 cells transformed with αDENV-U143 group I introns having either ΔN Bax or FL 3’ exons we qualitatively validated the effectiveness of each intron as a transgenic suppressive molecule. However, we expected that expression of a DCA-ΔN Bax fusion product following DENV targeting αDENV-U143- ΔN Bax would enhance the suppressive effect over the similar αDENV-U143-FL group I intron expressing the non-apoptotic DCA-FL fusion product. The levels of virus produced from cultures of C6/36 cells stably expressing either αDENV-U143-FL or αDENV-U143-ΔN Bax were determined using TCID_50_-immunofluorescence antibody assays following challenge with 0.1 MOI of each DENV serotype indicated (Figure 6; [10]). C6/36 cell lines stably expressing αDENV-U143-ΔN Bax consistently exhibited a large reduction of virus titer, as much as 5 logs, in comparison to the untransformed infection controls. Moreover, cultures transformed with αDENV-U143- ΔN Bax exhibited approximately 2.5 logs greater suppression than those transformed with αDENV-U143-FL (Figure 6), confirming enhancement of DENV suppression with ΔN Bax as the 3’ exon. Suppression of virus replication is evident regardless of DENV serotype targeted, or whether the DCV-IRES/mCherry configuration is present in the anti-DENV constructs. Minor decreases in viral titers observed for cells stably expressing the inactive anti-DENV group I intron ΔU143 or the constitutively expressed mCherry control construct may be attributed to interference from residual hygromycin [55].

### Expression of the αDENV-U143-ΔN intron in clonal cell populations leads to full suppression of DENV replication

While we were able to verify effective targeting and suppression of DENV in transformed hygromycin-selected C6/36 cells expressing our αDENV-U143 group I introns, antibiotic selection alone was not capable of eliminating all non-transformed cells resulting in some DENV infection in the cultures. We expected that cloned cell populations expressing αDENV-U143-ΔN Bax and αDENV-U143-FL would demonstrate significantly improved DENV suppression.

**Figure 7:**
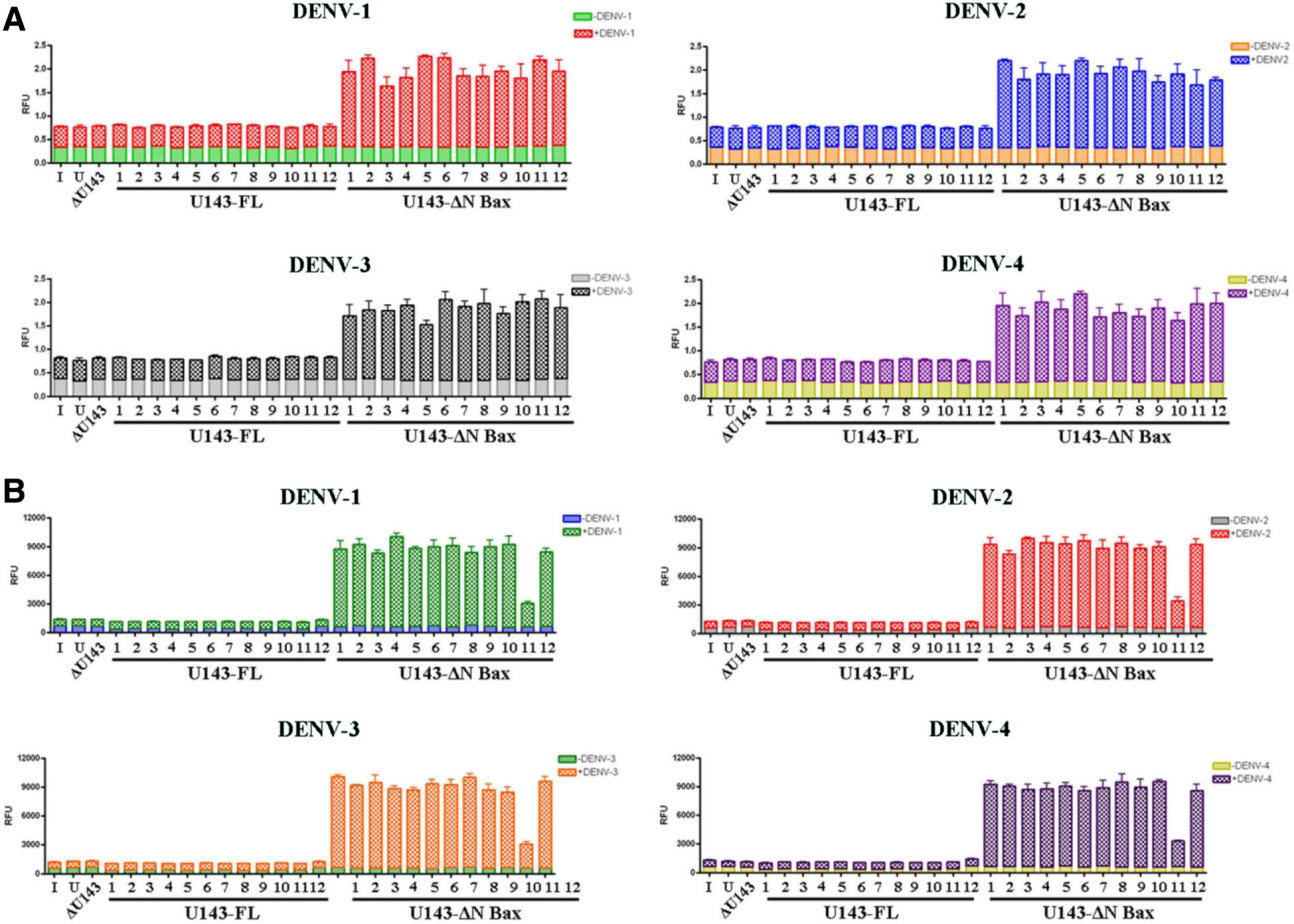
**αDENV**-**U143**–**ΔN Bax constructs effectively suppress DENV in clonal populations of transformed mosquito cells. A**. Clonal cell populations (labeled C-1 through C-11) were challenged with DENV-2 NGC (MOI 0.01). At 4 dpi cell supernatants were collected and saved for RT-PCR analysis. Following DENV-2 E protein antigen staining with antibody, micrographs were taken using the A1-R confocal microscope (Nikon). The figure displayed is an example of what is shown in Additional file 1: Figure S1. Green glowing cells, indicative of positive DENV E-2 staining, were observed only in cells actively replicating DENV. **B**. RT-PCR analysis of αDENV-U143-FL and αDENV-U143-ΔN Bax clonal cell populations. Supernatants from cell populations were collected prior to antibody staining of cells. RNAs were extracted as described in Methods. RT-PCR amplification was performed with primers to DCA and amplification products were separated on a 2% TAE agarose gel. Control RT-PCR experiments were performed with primers to actin to confirm RNA loading. Heterolgous primers to the intron- ΔN Bax segment of the construct were performed to confirm the presence of our anti-DENV effectors. The approximate sizes (in bases) of the RT-PCR products are indicated. Representative infected cell cultures are shown. **C**. αDENV-U143–ΔN Bax is capable of full DENV suppression. C6/36 clonal cell populations (designated C-1 through C-12) expressing αDENV-U13-FL or αDENV-U143–ΔN Bax were infected with each of the four DENV serotypes and TCID_50_-IFA analysis was performed as described in Methods. I-Wt = infected naïve C6/36 cells. I-mCh = DENV infected C6/36 cells transformed with a construct constitutively expressing the mCherry fluorescent marker. U = uninfected naïve C6/36 cells.

αDENV-U143-ΔN Bax and αDENV-U143-FL cell clones were established using limited dilution (Methods), challenged with DENV-2 NGC (MOI = 0.1), and analyzed for infectivity by fluorescence microscopy (Figure 7A and Additional file 2: Figure S2). All but one of the 12 αDENV-U143-ΔN Bax clones analyzed (C-11) displayed effective suppression of DENV-2 as opposed to 5 out of 12 for the αDENV-U143-FL transformed clones, clearly demonstrating an enhancing effect associated with including the proapoptotic ΔN Bax gene as a 3’ exon. These results illustrate the capabilities of this approach as a means of achieving complete suppression of viral infection when αDENV-U143-ΔN Bax is expressed in all cells.RT-PCR analysis of cloned cell supernatants (Figure 7B) confirmed our antibody assay results (see Figure 7A). RT-PCR of cell supernatants harvested from DENV infected cells at 4 dpi demonstrated that cloned cell populations expressing αDENV-U143-ΔN Bax (except C-11) are unable to support DENV replication as evidenced by the inability to detect DENV RNA.

To assess the effectiveness of these clones in suppressing all DENV serotypes we performed TCID_50_-IFA [17,18] at four days following infections of each αDENV-U143-FL and αDENV-U143-ΔN Bax cell clone with each DENV serotype (MOI = 0.01). As expected, quantitation of DENV replication suppression mirrored the fluorescence microscopy and RT-PCR assays for all DENV serotypes (Figure 7C). Total suppression of all DENV serotypes was observed in 11 of the 12 αDENV-U143-ΔN Bax clones as compared to only 5 of 12 for the αDENV-U143-FL cloned populations, providing additional confirmation of an enhancement of DENV suppression with proapoptotic ΔN Bax as the 3’ exon over FL. Suppression of DENV replication is evident regardless of serotype targeted.

The lone C6/36 clonal cell line stably expressing αDENV-U143-ΔN Bax that did not display full DENV suppression (C-11) still displayed a significant reduction in DENV titer, approximately 5 logs, in comparison to the infection control. This likely reflects that this was not a purely clonal population of cells or that the αDENV-U143-ΔN Bax was poorly expressed in these cells. We attribute the minor decreases in viral titers observed for cells stably expressing the inactive αDENV-ΔU143 or the constitutively expressed mCherry control construct to interference from residual hygromycin [55].

### ΑDENV-U143-ΔN Bax initiates apoptosis upon dengue virus infection

**Figure 8:**
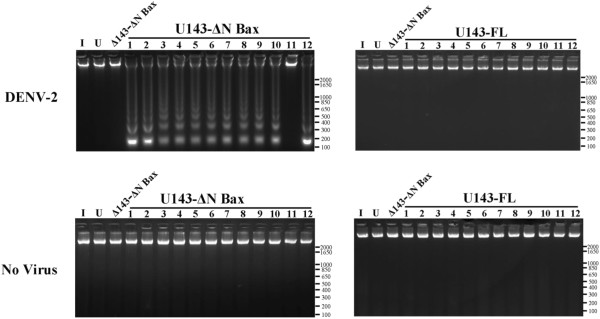
**Annexin V and Caspase 3 assays validate apoptosis by αDENV**-**ΔU143**-**ΔN Bax activation. A**. Promotion of the initial stages of apoptosis by αDENV-ΔU143-ΔN Bax activation. Clonal *Aedes albopictus* C6/36 cells transformed with αDENV -U143-FL, αDENV -ΔU143-ΔN Bax or αDENV -U143-ΔN Bax were challenged with either one of the four DENV serotypes. 1x10^6^ cells were stained with FITC conjugated Annexin V at 48 hpi (with DENV) and analyzed in a 96-well microtiter plate format per the manufacturer’s instructions (Cayman Chemical Company). Uninfected clonal and wild type C6/36 cells were also assayed as an additional negative control. Assays were performed in triplicate. Error bars indicate standard deviation of three independent experiments. **B**. Caspase-3 activation confirms the induction of apoptosis by the activation of Dual Targeting Intron Constructs. Clonal *Aedes albopictus* C6/36 cells transformed with αDENV -U143-FL, αDENV-ΔU143-ΔN Bax or αDENV -U143-ΔN Bax constructs were challenged with either one of the four DENV serotypes (MOI 0.1). 1x10^6^ cells were assessed for caspase 3 activity, an indication of apoptosis at 4d.pi (for DENV), and analyzed for caspase activity in a 96-well microtiter plate format per the manufacturer’s instructions (see Methods). Uninfected clonal and wild type C6/36 cells were also assayed as an additional negative control. Assays were performed in triplicate. Error bars indicate standard deviation of three independent experiments. I = infected naïve C6/36 cells. U = uninfected naïve C6/36 cells. ΔU143 = the anti-DENV group I intron (αDENV-ΔU143) possessing the inactive deletion mutation of the trans-splicing domain.

**Figure 9:**
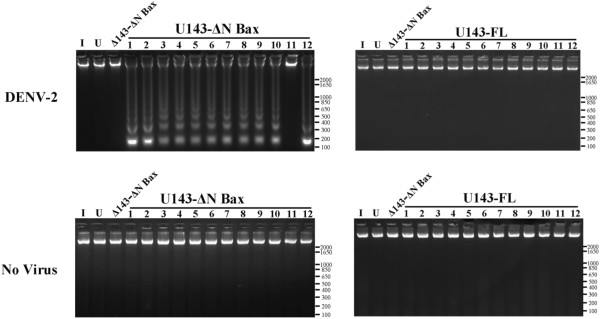
**DNA Fragmentation Assay: Clonal *Aedes albopictus *C6/ 36 cells transformed with αDENV -U143-FL, αDENV -ΔU143-ΔN Bax or αDENV-U143-ΔN Bax were each challenged with one of four known DENV serotypes (MOI 0.1).** At 4 dpi (for DENV) cells were pelleted, lysed, and analyzed as described in Methods. Uninfected clonal and wild type C6/36 cells were also assayed as an additional negative control.

While infection assays clearly demonstrated suppression of DENV infection and generation of the appropriate splice products, we needed to verify the improved effectiveness of the ΔN Bax 3’ exon resulted from induction of apoptosis in DENV infected cells expressing the αDENV-U143-ΔN Bax. We chose three assays, annexin V staining (Figure 8A), caspase 3 expression (Figure 8B), and DNA ladder production (Figure 9 and Additional file 3: Figure S3) to verify unambiguously the induction of all stages of apoptotic cell death as a response to DENV infection of cells expressing the αDENV-U143-ΔN Bax.

Annexin V-FITC assays [51,56,57] were performed to directly demonstrate the ability of the expressed DCA-ΔN Bax fusion proteins to initiate apoptosis. Following DENV challenge at 0.1 MOI with each serotype, 1×10^6^ transformed C6/36 clonal cells stably expressing αDENV-U143-ΔN Bax constructs were washed and stained with FITC-conjugated annexin V at 48 hours post infection and analyzed on a 96 well microtiter plate as described in Methods (Figure 8A). As a control, C6/36 cell lines stably expressing αDENV-U143-ΔN Bax constructs in the absence of virus demonstrated no annexin V staining, which also verified that the insertion of the UAA codon in the P9.0 helix of the group I intron effectively insures no expression of the ΔN Bax effector gene in the absence of splicing.Annexin V staining of cloned cells expressing the αDENV-U143-ΔN Bax linked to a DCV-IRES/mCherry reporter demonstrated approximately 3 fold activation of the initial stages of cellular apoptosis over untransformed control infected cells, and 2 fold greater induction of apoptosis over infected αDENV-U143-FL expressing cells. The induction of apoptosis occurred irrespective of the DENV serotype used as challenge (Figure 8A), with C-11 surprisingly exhibiting similar apoptosis levels compared to the 11 other cloned cell lines.As expected, neither wild type C6/36 or C6/36 cells transformed with a mCherry fluorescence marker displayed detectable annexin V-FITC staining (Figure 8A) comparable to αDENV-U143-ΔN Bax clonal lines following DENV infection. This result was also observed for cells stably expressing the inactive intron αDENV-ΔU143-ΔN Bax.

The activation of caspases (cysteinyl aspartate-specific proteases) is an important marker of the cell’s entry point into the apoptotic signaling cascade [58,59]. C6/36 cell clones expressing αDENV-U143-ΔN Bax linked to a DCV-IRES/mCherry configuration were challenged with each of the four DENV serotypes (MOI = 0.01) and analyzed at 4 d.p.i. for the progression of apoptosis by measuring caspase 3 activity in 96 well microtiter plates (Figure 8B) as described in Methods. As a control, C6/36 clonal cell lines stably expressing the αDENV-U143-ΔN Bax intron were assayed in the absence of virus to verify that stable expression of the intron itself does not trigger apoptosis, which again confirmed the effectiveness of the UAA codon in the P9.0 helix of the group I intron in preventing premature expression of the ΔN Bax effector gene.Our caspase-3 assays mirrored the annexin V staining results indicating apoptotic cell death in our DENV infected αDENV-U143-ΔN Bax transformed cell clones. Caspase-3 activity was detected in all DENV infected cloned cell populations irrespective of the serotype challenge, and at levels approximately 10 fold greater than control infected cells that did not possess an active anti-DENV intron. Once again, clone C-11 exhibited reduced caspase activity of approximately two fold greater than negative control infected cell lines. As expected, control wild type C6/36, C6/36 cells transformed with the mCherry fluorescence marker, and cells expressing the inactive αDENV-ΔU143-ΔN Bax displayed detectable caspase 3 activity following challenge with DENV (Figure 8B).

As a final demonstration of apoptotic activity we examined the characteristic degradation of nuclear DNA into nucleosomal units of approximately 180 bp in length [60]. DNA fragmentation analysis was performed as previously described [61] on infected αDENV-U143-ΔN Bax clonal cells infected with each of the four DENV serotypes (Figure 9 and Additional file 3: Figure S3). Briefly, at 5 d.p.i. cells were lysed, proteinase K and RNase A treated, and bands separated by electrophoresis on a 2% gel. As expected, infection of wild type C6/36 cells stably expressing the mCherry fluorescent marker or αDENV-U143-FL did not result in fragmentation of nuclear DNA. Similarly, clonal populations stably expressing αDENV-U143-ΔN Bax did not display observable DNA fragmentation in the absence of DENV infection, again confirming no premature expression of the ΔN Bax effector gene. Irrespective of the DENV serotype used, DNA fragmentation was evident in all αDENV-U143-ΔN Bax clonal cell populations tested except C-11, mirroring both TCID_50_ and Caspase 3 assay results.

## Discussion

This study examines the effectiveness of a constitutively expressed group I intron that targets and *trans*-splices conserved sequences and induces apoptotic cell death upon infection as a means of suppressing DENV virus infection of transformed mosquito cells. Group I *trans*-splicing introns have established potential for targeting RNA virus genomes in infected cells [18,32,33]. Previously we determined an optimal group I intron target sequence following an alignment of 98 instances of DENV that identified one highly conserved region positioned within the capsid coding sequence at nucleotides C131 to G151 [38,62]. These nucleotides are a part of the 5’-3’ CS domain of the DENV genome [18] that is essential for DENV replication [62]. We designed an anti-DENV group I *trans*-splicing intron, with firefly luciferase serving as the 3’ exon, and demonstrated its ability to effectively cleave at nucleotide position U143 [18]. We also demonstrated its ability to effectively *trans*-splice an infecting DENV-2 NGC when constitutively expressed as RNA in transformed C6/36 cells [18].

In this report we demonstrate the feasibility of using the U143 targeting group I intron, αDENV-U143, to catalyze *trans*-splicing of the 5’ CS region of DENV genomes to a 3’ ΔN Bax exon to induce apoptotic death of cells upon infection. A UAA stop codon inserted in the *trans*-splicing domain of the intron prevents premature expression of the ΔN Bax 3’ exon that would induce cell death in uninfected cells. Upon infection, αDENV-U143-ΔN Bax targeting and cleavage of DENV genomes at uracil 143 forms a chimeric mRNA that consists of the 5’ cap, 5′ UTR, 143 nucleotides of the DENV capsid (DCA) coding sequence, and the 3’ ΔN Bax exon. This chimeric RNA is capable of expressing a DCA-ΔN Bax fusion protein that induces apoptotic cell death precluding productive virus infection.

The strategy of targeting conserved sequences in the CS region of the genome cannot be considered immune to the evolution of escape mutations, but the extreme conservation of this region among all DENV, and even among Flaviviruses, suggests a markedly decreased potential for these mutations to develop. One obvious drawback to using these catalytic RNA molecules as simple genome degrading agents is that if the rate of virus replication exceeds the rate of group I intron catalytic suppression the evolution of escape mutants may be enhanced. An added level of insurance against the development of escape mutants should be achieved through the induction of cellular apoptotic pathways in response to DENV infection. Coupling the splicing activity of the group I intron to a death-upon-infection strategy insures that DENV replication rates do not exceed group I intron expression and catalytic rates, and should decrease the probability of generating escape mutants.

The use of a group I intron to induce cellular death upon infection has potential advantages over an RNAi suppression strategy since the length of conserved sequence necessary for group I intron targeting can be discontinuous as well as smaller than that required for RNAi-mediated responses. While successful RNAi responses in mosquitoes have been developed to directly target individual dengue serotype genomes [15,16,63-66], the RNAi approach may have difficulties targeting all serotypes simultaneously, and there is the possibility that escape mutants may amplify without restriction in response to the RNAi suppression.

The targeting and cleavage capability of our intron constructs was demonstrated with transient transfection assays in C6/36 mosquito cells challenged with infectious DENV. Sequencing analysis confirmed that the correct splice product was obtained, indicating proper targeting and site-specific cleavage of the DENV genome by the transiently expressed αDENV-U143 introns. Addition of the IRES/mCherry reporter configuration immediately downstream of the 3’ ΔN Bax exon did not appear to alter the *trans*-splicing capabilities of the intron, or affect the ability of the DCA-ΔN Bax resulting from the splice product to initiate apoptosis in DENV infected cells.

Our 5’-RLM-RACE results demonstrate that there is a 56 nt 5’ extension of RNA sequence in our anti-DENV group I intron resulting from expression by the A5c promoter that does not prohibit targeting and *trans*-splicing of DENV genomes (Additional file 1: Figure S1). However, we cannot rule out the possibility that an enhancement in anti-DENV group I intron activity could be achieved if the 56nt 5’ extension could be eliminated. This does not appear to be possible at this time since all RNA pol II promoters add a 5’ extension of considerable length due to the placement of their respective TSS, and elimination of this sequence typically results in greatly diminished RNA pol II promoter activity [67].

Expression and pro-apoptotic function of ΔN Bax is not inhibited by the 19 amino acids of the Dengue CA protein fused to its N-terminus. Expression and activity of ΔN Bax or DCA-ΔN Bax expressed in cells is not significantly different, and *trans*-splicing of the DENV RNA genome by αDENV-U143-ΔN Bax leads to the activation of cellular apoptosis as indicated by annexin V-FITC (Figure 8A), caspase 3 assays (Figure 8B), and DNA ladder analysis (Figure 9 and Additional file 3: Figure S3).

In regards to the difference between Annexin V and Caspase-3 assays with reference to the C-11 cell clone, several labs have successfully demonstrated that the early stages of apoptosis can be reversed [68-72]. The ability of U143-ΔN Bax mRNA to translocate out of the nucleus may be hampered, leading to a decrease in the number of DCA-ΔN Bax mRNA fusion molecules that are produced following DENV-U143-ΔN Bax targeting of DENV RNA in the cytoplasm of C-11 clonal cells. The diminished protein expression of DCA-ΔN Bax perturbs the progression of C-11 from the early stages of apoptosis (positive Annexin V staining) to the latter stages of apoptosis (negative Caspase 3 activity and DNA fragmentation). None of these assays indicate apoptotic cell death when the *trans*-splicing negative αDENV-ΔU143 or mCherry are expressed or when FL is used as the 3’ exon, confirming our results are a consequence of the presence of a *trans*-spliced RNA encoding the DCA-ΔN Bax. Other researchers have analyzed N-terminal epitope-tagged variants of tBax with little alteration in activity [40,73,74]. This is likely due to the fact that the C-terminal residues of Bax possess the pore forming function of this pro-apoptotic protein.

TCID_50_-IFA results demonstrate suppression of infectious virus production from our transformed and hygromycin selected cell lines upon challenge with each of the four serotypes (Figure 6). While we observe as much as a 5 log decrease in viral titer with each of the four serotypes targeted, the effector gene is even more potent than these uncloned, hygromycin-selected transformed cells demonstrate because these cultures necessarily produce hygromycin-resistant, non-transformed susceptible cells. Support for this reasoning is provided by the observation that removal of hygromycin selection results in a rapid recovery of virus susceptibility for our transformed cultures.In contrast, a greater antiviral effect was observed with transformed clonal cell populations in which every cell is confirmed to express the αDENV-U143-ΔN Bax intron by detection of the DCV-IRES expression of the mCherry marker from the same transcript (Figure 7). The enhanced DENV suppression observed for αDENV-U143-ΔN Bax vs. αDENV-U143-FL clones reflects a lack of dependency upon complete cleavage of all DENV genomes within infected cells expressing αDENV-U143-ΔN Bax due to the potency of the proapoptotic DCA-ΔN Bax product generated. Our results validate the utility of this single antiviral effector gene as a means for producing transgenic mosquitoes that will be refractory for DENV transmission.

Recently, a DENV-5 serotype has been identified in non-human primates from Malaysia that is characterized by a different antibody profile than the four known DENV serotypes [75]. This discovery will significantly impact vaccine development efforts, and may further enhance the attractiveness of anti-DENV transgenic mosquito strategies that can affect all serotypes. Although no sequence data is available for DENV-5 at the time of this submission, there is a high likelihood that the 5’-3’ CS domain will be conserved in this isolate as well, making it susceptible to our anti-DENV group I intron, U143.

We now have an anti-DENV group I intron that allows us to target at least four, and likely all five, DENV serotypes simultaneously. Targeting all serotypes with a single catalytic ribozyme or siRNAs eliminates the necessity to construct and test separate catalytic RNAs or siRNA molecules. However, unlike siRNA molecules that have been designed to target conserved regions of DENV in mammalian cells, the induction of cellular apoptosis by our αDENV- U143-ΔN Bax construct following DENV *trans*-splicing eliminates escape mutants that may evolve in the infected cell and prevents virus replication from overriding the catalytic activity of the anti-DENV group I intron.

These results foreshadow the potential efficacy of our U143-ΔN Bax constructs against DENV infection of transgenic mosquitoes expressing these antiviral effectors. Based on natural infection rates of midgut cells and the regenerative capabilities of midgut epithelia we do not expect that the loss of cells upon ingestion of a blood meal will have a significant impact on the survivability of the transgenic mosquitoes. This is a potential advantage in the dissemination of this transgene within the native population. Finally, our demonstration that the appended 56 nucleotide 5’ extension resulting from transcription of the U143 intron does not inhibit targeting or *trans*-splicing of the DENV RNA genome suggests to us that an antiviral group I intron construct capable of targeting multiple viruses simultaneously should be possible. For us the most likely virus candidates for such a dual targeting construct would be DENV and chikungunya viruses since these co-endemic pathogens have been shown to simultaneously infect humans and vector mosquitoes [76,77].

## Methods

### Cells, virus and antibody

*Ae. albopictus* C6/36 cells were obtained from ATCC, and maintained in Leibovitz’s L-15 media (Atlanta Biologicals) supplemented with 10% FBS (Atlanta Biologicals), 10% TPB (triptose phosphate broth; Invitrogen/Gibco), penicillin G (100 U/ml; Invitrogen/Gibco) and streptomycin (100 μU/ml; Invitrogen/Gibco). The C6/36 cells used in this study were maintained in a 28°C incubator and passaged every 4 days. For assays involving DENV infection, L-15 media supplemented with 2% FBS and 10% TPB were used. Viral stocks were prepared as previously described [17].

DENV sequence data for the four serotypes used in this study were obtained from NCBI, and comprise the following Genbank GenInfo identifiers: DENV type 1 Hawaii: DQ672564.1, DENV type 2 strain New Guinea C (NGC): AF038403.1, DENV type 3 strain ThD3 0010 87(strain H87): AY676352.1, DENV 4 strain DENV-4/SG/06K2270DK1/2005 (strain H241): GQ398256.1.

### Plasmid construction

The identity and integrity of all plasmids used were assured through sequencing and restriction analysis. All restriction enzymes were obtained from New England Bio Labs (NEB). See Additional file 4: Table S1 for sequences of all primers used.

Expression plasmids for analysis of ΔN Bax fusion protein activity were constructed from the *D. melanogaster* MT inducible promoter vector, pMT-V5-HisA (Invitrogen). Individual plasmids containing insertions of PCR EGFP (pMT-EGFP), full length Bax (pMT-Bax) transcript variant alpha (GenBank accession: NM-138761), or ΔN Bax ORF (pMT-ΔN Bax) were constructed by insertion of synthesized sequences (Bio Basic, Inc.) into *EcoRI*/*NotI* digested pMT-V5-HisA. Plasmid pMT-DCA was constructed by inserting the 5’ terminal fragment of the DENV-2 genome PCR amplified from pRS424-DENV-2 NGC [78] into the *EcoRI*/*XhoI* digested pMT-V5-HisA plasmid (Additional file 4: Table S1). The pMT-DCA-ΔN Bax fusion plasmid was constructed by insertion of the ΔN Bax sequence into the *XhoI*/*MluI* digested pMT-DCA vector.

The *Drosophila melanogaster* actin 5c (A5c) promoted U143 *trans*-splicing intron employed in this study was used previously to *trans*-splice DENV type 2-NGC targets to the firefly luciferase (FL) as the 3’ exon [18]. Our negative control for *trans*-splicing activity, ΔU143, was produced by removing the entire catalytic core [43], domains P4 through P6 of the U143 by PCR amplification with Platinum Taq polymerase (Invitrogen) using the forward and reverse primers listed in Additional file 4: Table S1 (pA5c-Δ U143-ΔN Bax). The PCR product was used to replace the catalytic core of the group I intron in U143 using the enzymes *MluI* and *XhoI* resulting in a control intron that lacked *trans*-splicing activity.

Anti-DENV group I introns U143 and ΔU143 constructs possessing the apoptotic ΔN Bax 3’ exon were assembled by insertion of a PCR amplified 243 nucleotide ΔN Bax gene (Additional file 1: Table S1; ΔN Bax) into the *XhoI*/*NotI* cleaved pA5c-U143-FL plasmid [18], replacing the FL 3’ exon to yield pA5c-ΔU143-ΔN Bax. Production of anti-DENV group I intron constructs possessing the DCV intergenic IRES site driving an mCherry fluorescent marker was achieved by subcloning DCV-mCherry from the corresponding U143-FL construct into pA5c U143-ΔN Bax using *XbaI* and *SacI* restriction sites ( [18]; Figure 2).

The cDNA plasmids encoding the predicted DENV-ΔN Bax *trans*-spliced products for 4 DENV serotypes were prepared by RT-PCR amplification of the DENV 5’ UTRs from each virus with *MluI* and *XhoI* tailed primers (Additional file 4: Table S1). The resulting PCR fragments were digested and ligated into pA5c- U143-ΔN Bax in place of the U143. These constructs are named pA5c-DENV1-ΔN Bax + ctrl, pA5c-DENV2-ΔN Bax + ctrl, pA5c-DENV3-ΔN Bax + ctrl, and pA5c-DENV4-ΔN Bax + ctrl (Additional file 4: Table S1).

### Reverse transcription-PCR of DENV-ΔN Bax splice products derived from cell culture

Extraction of RNA from DENV infected and uninfected cells were performed using the Qiashredder and RNeasy Mini kits (QIAGEN Inc., Valencia, CA, USA). Extracted RNA (5ug) was DNase treated using Turbo DNA-free DNase (Applied Biosystems/Ambion, Inc. Austin, TX USA). RT-PCR was performed using the SuperScript III One-Step RT-PCR kit (Invitrogen) [18] except the PCR reaction was carried out for 50 cycles. Plasmids expressing each serotype-specific DENV-ΔN Bax splice product were used as an RT-PCR size control.

### RT-PCR for the presence of DENV

This assay was performed as described above for the analysis of *trans*-spliced products except DENV virions were extracted from C6/36 cell supernatants (500μl) by Trizol extraction. DNase I treated RNAs were amplified using the Access Quick RT-PCR kit (Promega). Following cDNA synthesis the PCR amplification was carried out for 25 cycles with primers to DENV-2 E.

### 5’-RLM-RACE (RNA ligase mediated-rapid amplification of cDNA ends)

This assay was performed as described by the manufacturer (Invitrogen). Briefly, following extraction from cells, 5 μg of total RNA was dephosphorylated with Antarctic phosphatase (New England Biolabs) to eliminate the 5’ phosphates from truncated mRNA and non-mRNA. After phenol/chloroform extraction, the 5’-Cap structure is removed from the dephosphorylated mRNA present in the sample with tobacco acid pyrophosphatase (TAP) which is required for ligation to the 5’ Oligo specific primer (see Additional file 4: Table S1). Following another round of phenol/chloroform extraction, the 5’ primer was ligated to the 5’ end of the full-length, decapped mRNA using T4 RNA ligase. RT-PCR was then performed as described above using a forward primer specific to the ligated Oligo-specific primer (Additional file 4: Table S1), and a reverse primer that binds to the *trans*-splicing domain of the group I intron (U143 Rev, Additional file 4: Table S1). Amplified DNA was resolve on a 2% agarose gel. A 250bp band, the predicted size of the TSS-containing transcript, was extracted using the Wizard SV gel extraction kit (Promega), and TOPO-TA cloned (Invitrogen) for sequencing.

### Western blot analysis for ΔN Bax induction

C6/36 cells were transfected with 0.2 ug of pMT-EYFP as a transfection and induction marker and 1g of either pMT-DCA as a negative control for apoptosis, pMT-ΔN Bax, pMT-DCA-ΔN Bax, pMT-FMDV2A-ΔN Bax using Cellfectin transfection reagent (Invitrogen) per the manufacturers protocol. At 48 hours post-induction with copper sulfate, the cells were scraped from the bottom of the well and the entire suspension, including detached cells, was centrifuged at 1000XG for 10 minutes. For whole cell lysates, cell pellets were resuspended with Laemmli buffer containing the following protease and phosphatase inhibitors: 10mM benzamidine, 10 mM sodium fluoride, 100mM sodium vanadatephenylmethanesulphonylfluoride (1mM) (PMSF), 25 μg/mL leupeptin, 25 μg/mL aprotinin, and 25 μg/mL pepstatin. Whole cell lysates were sonicated, and protein concentrations were determined by optical density spectrophotometry at 280 nm on a Nanodrop ND-1000 spectrophotometer (Nanodrop Technologies Inc., Wilmington, DE) and an equal amount of each protein sample was loaded in each well. Whole cell lysates were separated via 10% SDS-PAGE, transferred to nitrocellulose filters, blocked in 5% skim milk in PBS, and incubated overnight with mouse monoclonal anti-Bax antibody, sensitive to the extreme C-terminus of ΔN Bax, (BD Biosciences Pharmagen, San Jose, CA) at a concentration of 1:150. Anti-mouse HRP conjugated secondary antibody (Amersham Biosciences, Piscataway, NJ) was incubated with the filter at concentration of 1:5000 for 1 hour. Actin was visualized using a goat polyclonal anti-actin (Santa Cruz Biotechnology, Santa Cruz, CA) at a concentration of 1:100. Anti-goat HRP conjugated secondary antibody (Santa Cruz Biotechnology) was incubated with the filter at concentration of 1:5000 for 1 hour. Specific bands were detected via chemiluminescence (SuperSignal West Dura, Pierce, Rockford, IL) and exposure to x-ray film. Films were scanned with a flatbed computer scanner.

### Amido black assay

The amido black assay was performed as previously described [50]. Triplicate wells of C6/36 cells were co-transfected with 0.3 μg of pMT-EYFP as a transfection marker and 0.6 μg of either pMT-DCA, pMT-ΔN Bax, pMT-FMDV2A-ΔN Bax or pMT-DCA-ΔN Bax, and analysis was performed at 6, 24, and 48 hours following CuSO_4_ induction. Cells were washed twice with 1× PBS (pH7.4), and fixed in 4% gluteraldehyde (Fisher Scientific) for 15 minutes. The gluteraldehyde was aspirated and 1 ml of 0.1% amido black staining solution was added [0.1 gram amido black 10B (C.I. 20470, Sigma-Aldrich), 7.5ml glacial acetic acid (Fisher Scientific), 20 ml 100% ethanol (Pharmco-Aaper, Shelbyville, KY), in 100ml with deionized water]. The plate was gently rocked for 30 minutes, each well washed twice with 1 ml 0.1 M acetate (pH4.5), and eluted with 1 ml 50 mM NaOH. Optical absorbency was read on a Nanodrop ND-1000 spectrophotometer at 620 nm and at 405 nm. The value obtained at 405 nm was subtracted from that obtained at 620 nm for the reading [50]. The value recorded for the pMT-DCA well, the apoptosis negative control, was set to 100% and all other readings adjusted by the same ratio to obtain a normalized reading. Data was analyzed using an ANOVA test with a Tukey’s post-test to compare all data sets within each time point for significance.

### Cytopathic effect (CPE) assay

This assay was performed as previously described [17]. Briefly, αDENV-U143-ΔN Bax transformed C6/36 cells were seeded at a density of 6 × 10^4^ cells/cm^2^ in T-25 flasks and incubated overnight at 28°C to allow attachment. Once attached cells were washed twice with plain L-15 media, and infected with the DENV serotype indicated (Figure. 5; 0.1 MOI). Micrographs were taken at 6 dpi with a Nikon E-600 inverted phase light microscope fitted with a Nikon DS Camera system at 20× magnification.

### Annexin V

Binding of annexin V to translocate the phospholipid phosphotidylserine (PS) allows for the detection and analysis of apoptotic cells [51,56,57]. These assays were performed using the *Annexin V FITC Assay Kit* as indicated by the manufacturer (Cayman Chemical Co.) with a few modifications. Briefly, C6/36 clonal cell lines stably expressing αDENV-U143-FL, αDENV-ΔU143-ΔN Bax or αDENV-U143-ΔN Bax ΔN Bax and wild type C6/36 cells were infected with each DENV serotype (MOI = 0.1). At 48 hpi 1 × 10^6^ clonal cells were scraped and placed in a well of a 96 well black opaque microtiter plate in triplicate for each clonal cell type assayed. FITC-annexin V microtiter plates were assayed for FITC-annexin V binding at 485 nm with the Spectra max M2 luminometer (Molecular Devices) and analyzed with Softmax Pro 5.4.5. Uninfected clonal and wild type C6/36 cells were also assayed as an additional negative control. Assays were performed in triplicate. Error bars indicate standard deviation of three independent experiments.

### Caspase 3 assay

Further validation of apoptosis induction was performed by assaying for increases in caspase-3 and other DEVD-specific protease activities using the *EnzChekCaspase*-*3 Assay Kit* #*2 Kit* (Life Technologies) as directed by the manufacturer. Briefly, C6/36 clonal cell lines stably expressing αDENV-U143-FL, αDENV-ΔU143-ΔN Bax or αDENV-U143-ΔN Bax and wild type C6/36 cells were infected with each DENV serotype (MOI = 0.1) and assayed for caspase 3 activity at 4 d.pi. 1×10^6^ clonal cells were lysed, cell debris was pelleted, and lysates were placed in a well of a 96 well black opaque microtiter plate in triplicate for each clonal cell type assayed. Following addition of the Z-DEVD–R110 substrate, microtiter plates were assayed for Caspase activity at 496 nm with the Spectra max M2 luminometer (Molecular Devices) and analyzed with Softmax Pro 5.4.5. Uninfected clonal and wild type C6/36 cells were also assayed as an additional negative control. Assays were performed in triplicate. Error bars indicate standard deviation of three independent experiments.

### DNA fragmentation assay

Clonal C6/36 cells stably expressing αDENV-U143-FL, αDENV-ΔU143-ΔN Bax or αDENV-U143-ΔN Bax and wild type C6/36 cells constructs were infected with one of four known DENV serotypes (MOI = 0.1) and assayed for DNA fragmentation as previously described [61]. Briefly, at 4 dpi cells were scraped, pelleted and lysed overnight at 50°C in lysis buffer [1.67 mg/ml Proteinase K, 10mM Tris (pH8.0), 100mM NaCl, 0.5% SDS 25mM EDTA]. Genomic DNA was extracted with 200 μl Phenol:Chloroform:IAA (25:24:1) and sodium acetate/ethanol precipitated. DNA pellets were resuspended in 20 μl TE buffer, RNase A treated (6.0 mg/ml) at 37°C for 3 hours, analyzed by 2% agarose gel electrophoresis at 5 v/cm and visualized under UV light. DNA fragmentation is demonstrated by the appearance of a DNA ladder-like pattern. Uninfected clonal and wild type C6/36 cells were also assayed as an additional negative control.

### TCID_50_-IFA analysis of dengue viruses

We used immunofluorescence detection of cell surface expressed DENV E protein in C6/36 cultures infected with serial 10 fold dilutions to assess DENV titer for all 4 serotypes as previously described [17]. 10 fold serial dilutions of infected C6/36 cell culture supernatants were harvested at 48 hpi and used as inoculum for 96 well plate cultures of naïve C6/36 cells. Plates were incubated for 4 days at 28°C without CO_2_, washed, fixed with acetone:DPBS (3:1), and stained with a primary DENV envelope (E) antibody (1:200) [79], followed by a biotinylated-streptavidin detection system conjugated with FITC (Amersham Biosciences, Piscataway, NJ). Wells displaying cellular fluorescence were scored as positive for DENV infection. The number of positive wells were counted and the virus titers calculated according to Karber’s method [80]. The deletion mutation of the *trans*-splicing domain (αDENV -ΔU143) is designed to knock out trans-splicing function, providing a negative control [81]. TCID_50_-IFA analysis of clonal cell populations expressing αDENV -U143-FL or αDENV -U143-ΔN Bax constructs was performed in this manner.

### Establishment of clonal cell populations

Clonal cell populations were produced as previously described [65]. Briefly, C6/36 cells stably expressing αDENV -U143-FL, αDENV -ΔU143-ΔN Bax or αDENV -U143-ΔN Bax were grown to 4 × 10^2^ cells/cm^2^ and then diluted to 0.2 cells/cm^2^. 100ul of this cell suspension was placed in each of a 96 well plate and grown to confluency. Twelve wells of each plate were scraped and transferred to individual wells of a 24 well plate. Once confluent, cells were then transferred to a 12 well plate, then a 6 well plate, and lastly T-25 flasks. At each transfer step cells were maintained with 1mL L-15 complete media supplemented with 100ug/mL hygromycin. In order to guarantee clonability 3 cloning cycles were carried out.

## Competing interests

The authors declare that they have no competing interests.

## Authors’ contributions

JRC engineered the αDENV-U143-ΔN Bax constructs possessing the IRES mCherry configuration. JRC also performed all final cell culture analysis of the αDENV-constructs including apoptosis and RT-PCR analyses, and produced all clonal cell lines used in this study. JHK engineered the pMT promoted plasmids, and produced the original αDENV-U143-ΔN Bax constructs used in all analysis. JAD performed 5’ RACE analysis. CAK propagated and maintained all virus stocks. KMH and SH performed initial TCID50-IFA and RT-PCR analysis. TSF maintained all cell cultures and established transformed cell lines. MJF developed the overall concept, secured support, provided research facilities, and was responsible for managing all aspects of the research. This manuscript was prepared by JRC and MJF, with editorial contributions from JHK, JAD, TSF, and CHK. All authors read and approved the final manuscript.

## Supplementary Material

Additional file 1: Figure S1 5’ RACE transcription start site (TSS) analysis. A. The predicted sequences of the Actin 5c promoter with the putative TSS [82] and αDENV-U143 as transcribed in the cell. The adenine depicting the end of the putative TSS is green, and the 3’ end of the promoter is red. The IGS and EGS located 6 nucleotides downstream of the 3’ end of the A5c promoter are labeled and underlined. Nucleic acids are numbered in relation to their position downstream from the TSS (+1). B. RT-PCR products were resolved by 2% agarose gel electrophoresis as described in Methods. The RACE product amplified from αDENV-U143-ΔN Bax was approximately 250 bp as indicated by the asterisk. L = standard ladder. C. Alignment resulting from sequencing eleven isolates following TOPO cloning of the 250 bp fragment displayed in Additional file 1: Figure S1B. The putative TSS and actual TSS of each colony isolate of 11 are aligned. Complete homology is shown in yellow, partial consensus in blue. Click here for file

Additional file 2: Figure S2 Clonal cell populations (labeled C-1 through C-11) were challenged with DENV-2 NGC (MOI = 0.01). At 4 dpi cell supernatants were collected and saved for RT-PCR analysis (see 8b). Following DENV-2 E protein antigen staining with antibody, micrographs were taken using the A1-R confocal microscope (Nikon). I = infected, U = uninfected. Click here for file

Additional file 3: Figure S3 Clonal cell populations stably expressing αDENV -U143-FL, αDENV -ΔU143-ΔN Bax or αDENV-U143-ΔN Bax (labeled C-1 through C-11) were challenged with DENV-1, DENV-2 (see Figure 9) DENV-3, or DENV-4 (MOI = 0.1). At 4dpi analysis of DNA fragmentation was performed as described in Methods. I = infected, U = uninfected. Click here for file

Additional file 4: Table S1 Primers used for plasmid construction and 5’ RLM-RACE. Listed are the forward and reverse primer sets used to produce the PCR fragments and 5’RLM-RACE analysis. Lowercase nucleic acids indicate restriction site. See Methods for description of vector constructs. Click here for file

## References

[B1] James AA Gene drive systems in mosquitoes: rules of the road Trends Parasitol 2005 21 64 67 15664528 10.1016/j.pt.2004.11.004

[B2] Sinkins SP Gould F Gene drive systems for insect disease vectors Nat Rev Genet 2006 7 427 435 16682981 10.1038/nrg1870

[B3] Clyde K Kyle JL Harris E Recent advances in deciphering viral and host determinants of dengue virus replication and pathogenesis J Virol 2006 80 11418 11431 16928749 10.1128/JVI.01257-06 PMC1642597

[B4] Bhatt S Gething PW Brady OJ Messina JP Farlow AW Moyes CL Drake JM Brownstein JS Hoen AG Sankoh O Myers MF George DB Jaenisch T Wint GR Simmons CP Scott TW Farrar JJ Hay SI The global distribution and burden of dengue Nature 2013 496 504 507 23563266 10.1038/nature12060 PMC3651993

[B5] Rai MA Epidemic: Control of dengue fever in Pakistan Nature 2011 479 41 22051664 10.1038/479041d

[B6] Maddox N Time to prepare for dengue testing? Florida Bureau of Labs is already on the job MLO Med Lab Obs 2011 43 44 45 21639009

[B7] Graham AS Pruszynski CA Hribar LJ DeMay DJ Tambasco AN Hartley AE Fussell EM Michael SF Isern S Mosquito-associated dengue virus, Key West, Florida, USA, 2010 Emerg Infect Dis 2011 17 2074 2075 22099104 10.3201/eid1711.110419 PMC3310564

[B8] Radke EG Gregory CJ Kintziger KW Sauber-Schatz EK Hunsperger EA Gallagher GR Barber JM Biggerstaff BJ Stanek DR Tomashek KM Blackmore CG Dengue outbreak in Key West, Florida, USA, 2009 Emerg Infect Dis 2012 18 135 137 22257471 10.3201/eid1801.110130 PMC3310087

[B9] Fact Sheet [http://www.who.int/mediacentre/factsheets/fs117/en/]

[B10] Frank C Hohle M Stark K Lawrence J More reasons to dread rain on vacation? Dengue fever in 42 German and United Kingdom Madeira tourists during autumn 2012 Euro Surveill 2013 18 20446 23594519 10.2807/1560-7917.es2013.18.14.20446

[B11] Wilder-Smith A Dengue in international travelers: quo vadis? J Travel Med 2013 20 341 343 24165378 10.1111/jtm.12063

[B12] Schirmer PL Lucero-Obusan CA Benoit SR Santiago LM Stanek D Dey A Martinez M Oda G Holodniy M Dengue surveillance in Veterans Affairs healthcare facilities, 2007–2010 PLoS Negl Trop Dis 2013 7 e2040 23516642 10.1371/journal.pntd.0002040 PMC3597482

[B13] Torres-Galicia I Cortes-Poza D Becker I [Dengue in Mexico: an analysis of two decades] Gac Med Mex 2014 150 122 127 24603992

[B14] Bisset JA Marin R Rodriguez MM Severson DW Ricardo Y French L Diaz M Perez O Insecticide resistance in two Aedes aegypti (Diptera: Culicidae) strains from Costa Rica J Med Entomol 2013 50 352 361 23540124 10.1603/me12064

[B15] Olson KE Higgs S Gaines PJ Powers AM Davis BS Kamrud KI Carlson JO Blair CD Beaty BJ Genetically engineered resistance to dengue-2 virus transmission in mosquitoes Science 1996 272 884 886 8629025 10.1126/science.272.5263.884

[B16] Franz AW Sanchez-Vargas I Adelman ZN Blair CD Beaty BJ James AA Olson KE Engineering RNA interference-based resistance to dengue virus type 2 in genetically modified Aedes aegypti Proc Natl Acad Sci U S A 2006 103 4198 4203 16537508 10.1073/pnas.0600479103 PMC1449670

[B17] Nawtaisong P Keith J Fraser T Balaraman V Kolokoltsov A Davey RA Higgs S Mohammed A Rongsriyam Y Komalamisra N Fraser MJ Jr Effective suppression of Dengue fever virus in mosquito cell cultures using retroviral transduction of hammerhead ribozymes targeting the viral genome Virol J 2009 6 73 19497123 10.1186/1743-422X-6-73 PMC2704196

[B18] Carter JR Keith JH Barde PV Fraser TS Fraser MJ Jr Targeting of highly conserved Dengue virus sequences with anti-Dengue virus trans-splicing group I introns BMC Mol Biol 2010 11 84 21078188 10.1186/1471-2199-11-84 PMC3000392

[B19] Ayre BG Kohler U Goodman HM Haseloff J Design of highly specific cytotoxins by using trans-splicing ribozymes Proc Natl Acad Sci U S A 1999 96 3507 3512 10097066 10.1073/pnas.96.7.3507 PMC22323

[B20] Byun J Lan N Long M Sullenger BA Efficient and specific repair of sickle beta-globin RNA by trans-splicing ribozymes RNA 2003 9 1254 1263 13130139 10.1261/rna.5450203 PMC1370489

[B21] Jung HS Kwon BS Lee SW Tumor-specific gene delivery using RNA-targeting Tetrahymena group I intron Biotechnol Lett 2005 27 567 574 15973491 10.1007/s10529-005-2883-6

[B22] Kastanos E Hjiantoniou E Phylactou LA Restoration of protein synthesis in pancreatic cancer cells by trans-splicing ribozymes Biochem Biophys Res Commun 2004 322 930 934 15336553 10.1016/j.bbrc.2004.07.203

[B23] Waterston RH Lindblad-Toh K Birney E Rogers J Abril JF Agarwal P Agarwala R Ainscough R Alexandersson M An P Antonarakis SE Attwood J Baertsch R Bailey J Barlow K Beck S Berry E Birren B Bloom T Bork P Botcherby M Bray N Brent MR Brown DG Brown SD Bult C Burton J Butler J Campbell RD Carninci P Initial sequencing and comparative analysis of the mouse genome Nature 2002 420 520 562 12466850 10.1038/nature01262

[B24] Ryu KJ Lee SW Identification of the most accessible sites to ribozymes on the hepatitis C virus internal ribosome entry site J Biochem Mol Biol 2003 36 538 544 14659071 10.5483/bmbrep.2003.36.6.538

[B25] Ryu KJ Lee SW Comparative analysis of intracellular trans-splicing ribozyme activity against hepatitis C virus internal ribosome entry site J Microbiol 2004 42 361 364 15650696

[B26] Sullenger BA Cech TR Ribozyme-mediated repair of defective mRNA by targeted, trans-splicing Nature 1994 371 619 622 7935797 10.1038/371619a0

[B27] Lander ES Linton LM Birren B Nusbaum C Zody MC Baldwin J Devon K Dewar K Doyle M FitzHugh W Funke R Gage D Harris K Heaford A Howland J Kann L Lehoczky J LeVine R McEwan P McKernan K Meldrim J Mesirov JP Miranda C Morris W Naylor J Raymond C Rosetti M Santos R Sheridan A Sougnez C Initial sequencing and analysis of the human genome Nature 2001 409 860 921 11237011 10.1038/35057062

[B28] Jeong JS Lee SW Hong SH Lee YJ Jung HI Cho KS Seo HH Lee SJ Park S Song MS Kim CM Kim IH Antitumor effects of systemically delivered adenovirus harboring trans-splicing ribozyme in intrahepatic colon cancer mouse model Clin Cancer Res 2008 14 281 290 18172280 10.1158/1078-0432.CCR-07-1524

[B29] Kwon BS Jung HS Song MS Cho KS Kim SC Kimm K Jeong JS Kim IH Lee SW Specific regression of human cancer cells by ribozyme-mediated targeted replacement of tumor-specific transcript Mol Ther 2005 12 824 834 16040278 10.1016/j.ymthe.2005.06.096

[B30] Watanabe T Sullenger BA Induction of wild-type p53 activity in human cancer cells by ribozymes that repair mutant p53 transcripts Proc Natl Acad Sci U S A 2000 97 8490 8494 10890910 10.1073/pnas.150104097 PMC26975

[B31] Rogers CS Vanoye CG Sullenger BA George AL Jr Functional repair of a mutant chloride channel using a trans-splicing ribozyme J Clin Invest 2002 110 1783 1789 12488428 10.1172/JCI200216481 PMC151654

[B32] Kohler U Ayre BG Goodman HM Haseloff J Trans-splicing ribozymes for targeted gene delivery J Mol Biol 1999 285 1935 1950 9925776 10.1006/jmbi.1998.2447

[B33] Ryu KJ Kim JH Lee SW Ribozyme-mediated selective induction of new gene activity in hepatitis C virus internal ribosome entry site-expressing cells by targeted trans-splicing Mol Ther 2003 7 386 395 12668134 10.1016/s1525-0016(02)00063-1

[B34] Holden KL Stein DA Pierson TC Ahmed AA Clyde K Iversen PL Harris E Inhibition of dengue virus translation and RNA synthesis by a morpholino oligomer targeted to the top of the terminal 3′ stem-loop structure Virology 2006 344 439 452 16214197 10.1016/j.virol.2005.08.034

[B35] Kinney RM Huang CY Rose BC Kroeker AD Dreher TW Iversen PL Stein DA Inhibition of dengue virus serotypes 1 to 4 in vero cell cultures with morpholino oligomers J Virol 2005 79 5116 5128 15795296 10.1128/JVI.79.8.5116-5128.2005 PMC1069583

[B36] Zeng L Falgout B Markoff L Identification of specific nucleotide sequences within the conserved 3′-SL in the dengue type 2 virus genome required for replication J Virol 1998 72 7510 7522 9696848 10.1128/jvi.72.9.7510-7522.1998 PMC109990

[B37] Hahn CS Hahn YS Rice CM Lee E Dalgarno L Strauss EG Strauss JH Conserved elements in the 3′ untranslated region of flavivirus RNAs and potential cyclization sequences J Mol Biol 1987 198 33 41 2828633 10.1016/0022-2836(87)90455-4

[B38] Alvarez DE Lodeiro MF Luduena SJ Pietrasanta LI Gamarnik AV Long-range RNA-RNA interactions circularize the dengue virus genome J Virol 2005 79 6631 6643 15890901 10.1128/JVI.79.11.6631-6643.2005 PMC1112138

[B39] Markoff L 5′- and 3′-noncoding regions in flavivirus RNA Adv Virus Res 2003 59 177 228 14696330 10.1016/S0065-3527(03)59006-6 PMC7119107

[B40] Usui K Saijo Y Narumi K Koyama S Maemondo M Kikuchi T Tazawa R Hagiwara K Ishibashi Y Ohta S Nukiwa T N-terminal deletion augments the cell-death-inducing activity of BAX in adenoviral gene delivery to nonsmall cell lung cancers Oncogene 2003 22 2655 2663 12730679 10.1038/sj.onc.1206331

[B41] Cech TR RNA editing: world’s smallest introns? Cell 1991 64 667 669 1997201 10.1016/0092-8674(91)90494-j

[B42] Bell MA Sinha J Johnson AK Testa SM Enhancing the second step of the trans excision-splicing reaction of a group I ribozyme by exploiting P9.0 and P10 for intermolecular recognition Biochemistry 2004 43 4323 4331 15065876 10.1021/bi035874n

[B43] Cech TR Self-splicing of group I introns Annu Rev Biochem 1990 59 543 568 2197983 10.1146/annurev.bi.59.070190.002551

[B44] Strobel SA Cech TR Tertiary interactions with the internal guide sequence mediate docking of the P1 helix into the catalytic core of the Tetrahymena ribozyme Biochemistry 1993 32 13593 13604 7504953 10.1021/bi00212a027

[B45] Campbell TB Cech TR Mutations in the Tetrahymena ribozyme internal guide sequence: effects on docking of the P1 helix into the catalytic core and correlation with catalytic activity Biochemistry 1996 35 11493 11502 8784205 10.1021/bi960510z

[B46] Guo F Cech TR In vivo selection of better self-splicing introns in Escherichia coli: the role of the P1 extension helix of the Tetrahymena intron RNA 2002 8 647 658 12022231 10.1017/s1355838202029011 PMC1370285

[B47] Doherty EA Batey RT Masquida B Doudna JA A universal mode of helix packing in RNA Nat Struct Biol 2001 8 339 343 11276255 10.1038/86221

[B48] Carter JR Fraser TS Fraser MJ Jr Examining the relative activity of several dicistrovirus intergenic internal ribosome entry site elements in uninfected insect and mammalian cell lines J Gen Virol 2008 89 3150 3155 19008405 10.1099/vir.0.2008/003921-0

[B49] Hobbs JA Hommel-Berrey G Brahmi Z Requirement of caspase-3 for efficient apoptosis induction and caspase-7 activation but not viral replication or cell rounding in cells infected with vesicular stomatitis virus Hum Immunol 2003 64 82 92 12507817 10.1016/s0198-8859(02)00702-4

[B50] Schulz J Dettlaff S Fritzsche U Harms U Schiebel H Derer W Fusenig NE Hulsen A Bohm M The amido black assay: a simple and quantitative multipurpose test of adhesion, proliferation, and cytotoxicity in microplate cultures of keratinocytes (HaCaT) and other cell types growing adherently or in suspension J Immunol Methods 1994 167 1 13 7508474 10.1016/0022-1759(94)90069-8

[B51] Muhlenfeld K Langner A Biotransformation and toxicity of the lipoxygenase inhibitor 2-hydroxy-5-methyllaurophenone oxime (FLM 5011) on Hep G2 cells Arch Pharm (Weinheim) 1998 331 259 261 9747183 10.1002/(sici)1521-4184(199807)331:7/8<259::aid-ardp259>3.0.co;2-7

[B52] Cavin Perier R Junier T Bucher P The Eukaryotic Promoter Database EPD Nucleic Acids Res 1998 26 353 357 9399872 10.1093/nar/26.1.353 PMC147208

[B53] Suitor EC Jr Paul FJ Syncytia formation of mosquito cell cultures mediated by type 2 dengue virus Virology 1969 38 482 485 5799585 10.1016/0042-6822(69)90162-7

[B54] Barth OM Schatzmayr HG Brazilian dengue virus type 1 replication in mosquito cell cultures Mem Inst Oswaldo Cruz 1992 87 1 7 1308543 10.1590/s0074-02761992000100001

[B55] Lacal JC Vazquez D Fernandez-Sousa JM Carrasco L Antibiotics that specifically block translation in virus-infected cells J Antibiot (Tokyo) 1980 33 441 446 6251018 10.7164/antibiotics.33.441

[B56] Koopman G Reutelingsperger CP Kuijten GA Keehnen RM Pals ST van Oers MH Annexin V for flow cytometric detection of phosphatidylserine expression on B cells undergoing apoptosis Blood 1994 84 1415 1420 8068938

[B57] Martin SJ Reutelingsperger CP McGahon AJ Rader JA van Schie RC LaFace DM Green DR Early redistribution of plasma membrane phosphatidylserine is a general feature of apoptosis regardless of the initiating stimulus: inhibition by overexpression of Bcl-2 and Abl J Exp Med 1995 182 1545 1556 7595224 10.1084/jem.182.5.1545 PMC2192182

[B58] Vermeulen K Berneman ZN Van Bockstaele DR Cell cycle and apoptosis Cell Prolif 2003 36 165 175 12814432 10.1046/j.1365-2184.2003.00267.x PMC6496173

[B59] Vermeulen K Van Bockstaele DR Berneman ZN The cell cycle: a review of regulation, deregulation and therapeutic targets in cancer Cell Prolif 2003 36 131 149 12814430 10.1046/j.1365-2184.2003.00266.x PMC6496723

[B60] Coupe SA Watson LM Ryan DJ Pinkney TT Eason JR Molecular analysis of programmed cell death during senescence in Arabidopsis thaliana and Brassica oleracea: cloning broccoli LSD1, Bax inhibitor and serine palmitoyltransferase homologues J Exp Bot 2004 55 59 68 14645391 10.1093/jxb/erh018

[B61] Wang H Blair CD Olson KE Clem RJ Effects of inducing or inhibiting apoptosis on Sindbis virus replication in mosquito cells J Gen Virol 2008 89 2651 2661 18931060 10.1099/vir.0.2008/005314-0 PMC2603079

[B62] Alvarez DE Filomatori CV Gamarnik AV Functional analysis of dengue virus cyclization sequences located at the 5′ and 3′UTRs Virology 2008 375 223 235 18289628 10.1016/j.virol.2008.01.014

[B63] Caplen NJ Zheng Z Falgout B Morgan RA Inhibition of viral gene expression and replication in mosquito cells by dsRNA-triggered RNA interference Mol Ther 2002 6 243 251 12161191 10.1006/mthe.2002.0652

[B64] Adelman ZN Blair CD Carlson JO Beaty BJ Olson KE Sindbis virus-induced silencing of dengue viruses in mosquitoes Insect Mol Biol 2001 10 265 273 11437918 10.1046/j.1365-2583.2001.00267.x

[B65] Adelman ZN Sanchez-Vargas I Travanty EA Carlson JO Beaty BJ Blair CD Olson KE RNA silencing of dengue virus type 2 replication in transformed C6/36 mosquito cells transcribing an inverted-repeat RNA derived from the virus genome J Virol 2002 76 12925 12933 12438618 10.1128/JVI.76.24.12925-12933.2002 PMC136701

[B66] Adelman ZN Jasinskiene N Vally KJ Peek C Travanty EA Olson KE Brown SE Stephens JL Knudson DL Coates CJ James AA Formation and loss of large, unstable tandem arrays of the piggyBac transposable element in the yellow fever mosquito, Aedes aegypti Transgenic Res 2004 13 411 425 15587266 10.1007/s11248-004-6067-2

[B67] Smale ST Kadonaga JT The RNA polymerase II core promoter Annu Rev Biochem 2003 72 449 479 12651739 10.1146/annurev.biochem.72.121801.161520

[B68] Tang HL Tang HM Mak KH Hu S Wang SS Wong KM Wong CS Wu HY Law HT Liu K Talbot CC Jr Lau WK Montell DJ Fung MC Cell survival, DNA damage, and oncogenic transformation after a transient and reversible apoptotic response Mol Biol Cell 2012 23 2240 2252 22535522 10.1091/mbc.E11-11-0926 PMC3374744

[B69] Geske FJ Lieberman R Strange R Gerschenson LE Early stages of p53-induced apoptosis are reversible Cell Death Differ 2001 8 182 191 11313720 10.1038/sj.cdd.4400786

[B70] Blankenberg FG In vivo imaging of apoptosis Cancer Biol Ther 2008 7 1525 1532 18836289 10.4161/cbt.7.10.6934

[B71] O’Brien IE Murray BG Baguley BC Morris BA Ferguson IB Major changes in chromatin condensation suggest the presence of an apoptotic pathway in plant cells Exp Cell Res 1998 241 46 54 9633512 10.1006/excr.1998.4036

[B72] Balasubramanian K Mirnikjoo B Schroit AJ Regulated externalization of phosphatidylserine at the cell surface: implications for apoptosis J Biol Chem 2007 282 18357 18364 17470427 10.1074/jbc.M700202200

[B73] Toyota H Kondo S Kyo S Mizuguchi J Enforced expression of a truncated form of Bax-alpha (tBax) driven by human telomerase reverse transcriptase (hTERT) promoter sensitizes tumor cells to chemotherapeutic agents or tumor necrosis factor-related apoptosis-inducing ligand (TRAIL) Anticancer Res 2006 26 99 105 16475685

[B74] Toyota H Yanase N Yoshimoto T Moriyama M Sudo T Mizuguchi J Calpain-induced Bax-cleavage product is a more potent inducer of apoptotic cell death than wild-type Bax Cancer Lett 2003 189 221 230 12490315 10.1016/s0304-3835(02)00552-9

[B75] Normile D Tropical medicine. Surprising new dengue virus throws a spanner in disease control efforts Science 2013 342 415 24159024 10.1126/science.342.6157.415

[B76] Myers RM Carey DE Concurrent isolation from patient of two arboviruses, Chikungunya and dengue type 2 Science 1967 157 1307 1308 6038994 10.1126/science.157.3794.1307

[B77] Leroy EM Nkoghe D Ollomo B Nze-Nkogue C Becquart P Grard G Pourrut X Charrel R Moureau G Ndjoyi-Mbiguino A De-Lamballerie X Concurrent chikungunya and dengue virus infections during simultaneous outbreaks, Gabon, 2007 Emerg Infect Dis 2009 15 591 593 19331740 10.3201/eid1504.080664 PMC2671412

[B78] Polo S Ketner G Levis R Falgout B Infectious RNA transcripts from full-length dengue virus type 2 cDNA clones made in yeast J Virol 1997 71 5366 5374 9188607 10.1128/jvi.71.7.5366-5374.1997 PMC191775

[B79] Henchal EA McCown JM Burke DS Seguin MC Brandt WE Epitopic analysis of antigenic determinants on the surface of dengue-2 virions using monoclonal antibodies Am J Trop Med Hyg 1985 34 162 169 2578750 10.4269/ajtmh.1985.34.162

[B80] Karber G 50% end-point calculation Arch Exp Pathol Pharmk 1931 162 480 483

[B81] Johnson TH Tijerina P Chadee AB Herschlag D Russell R Structural specificity conferred by a group I RNA peripheral element Proc Natl Acad Sci U S A 2005 102 10176 10181 16009943 10.1073/pnas.0501498102 PMC1177367

[B82] Abeel T Saeys Y Rouze P Van de Peer Y ProSOM: core promoter prediction based on unsupervised clustering of DNA physical profiles Bioinformatics 2008 24 i24 i31 18586720 10.1093/bioinformatics/btn172 PMC2718650

