# Supplementary figures and images for: Effective suppression of Dengue virus using a novel group-I intron that induces apoptotic cell death upon infection through conditional expression of the Bax C-terminal domain
Source: Virol J. 2014 Jun 13;11:111. doi: 10.1186/1743-422X-11-111 (PMC4104402; doi:10.1186/1743-422X-11-111)

## Slide 1
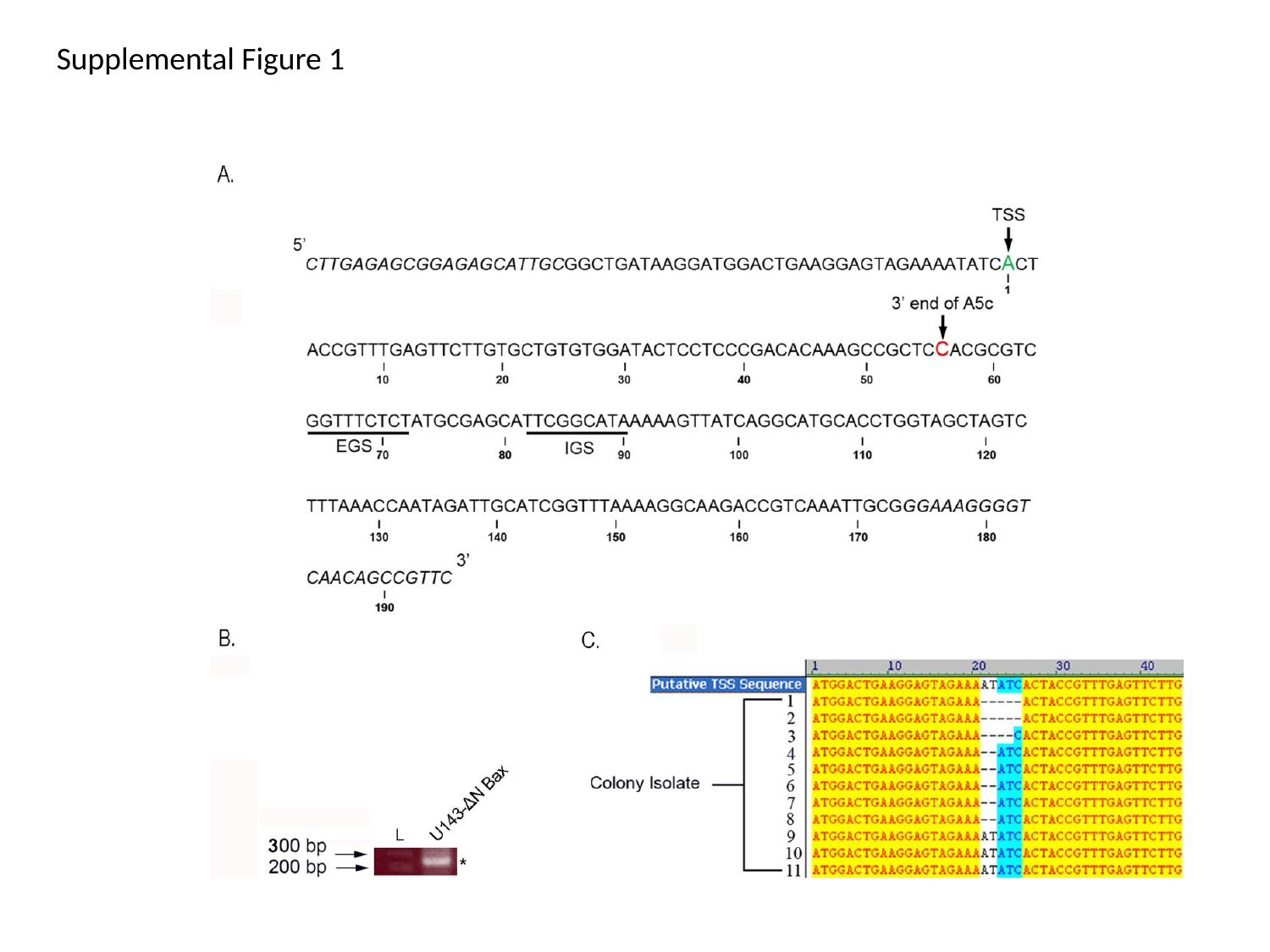

Supplemental Figure 1

Supplement: Additional file 1: Figure S1 — 5’ RACE transcription start site (TSS) analysis. A. The predicted sequences of the Actin 5c promoter with the putative TSS [82] and αDENV-U143 as transcribed in the cell. The adenine depicting the end of the putative TSS is green, and the 3’ end of the promoter is red. The IGS and EGS located 6 nucleotides downstream of the 3’ end of the A5c promoter are labeled and underlined. Nucleic acids are numbered in relation to their position downstream from the TSS (+1). B. RT-PCR products were resolved by 2% agarose gel electrophoresis as described in Methods. The RACE product amplified from αDENV-U143-ΔN Bax was approximately 250 bp as indicated by the asterisk. L = standard ladder. C. Alignment resulting from sequencing eleven isolates following TOPO cloning of the 250 bp fragment displayed in Additional file 1: Figure S1B. The putative TSS and actual TSS of each colony isolate of 11 are aligned. Complete homology is shown in yellow, partial consensus in blue. [file 1743-422X-11-111-S1.pptx]

## Slide 1
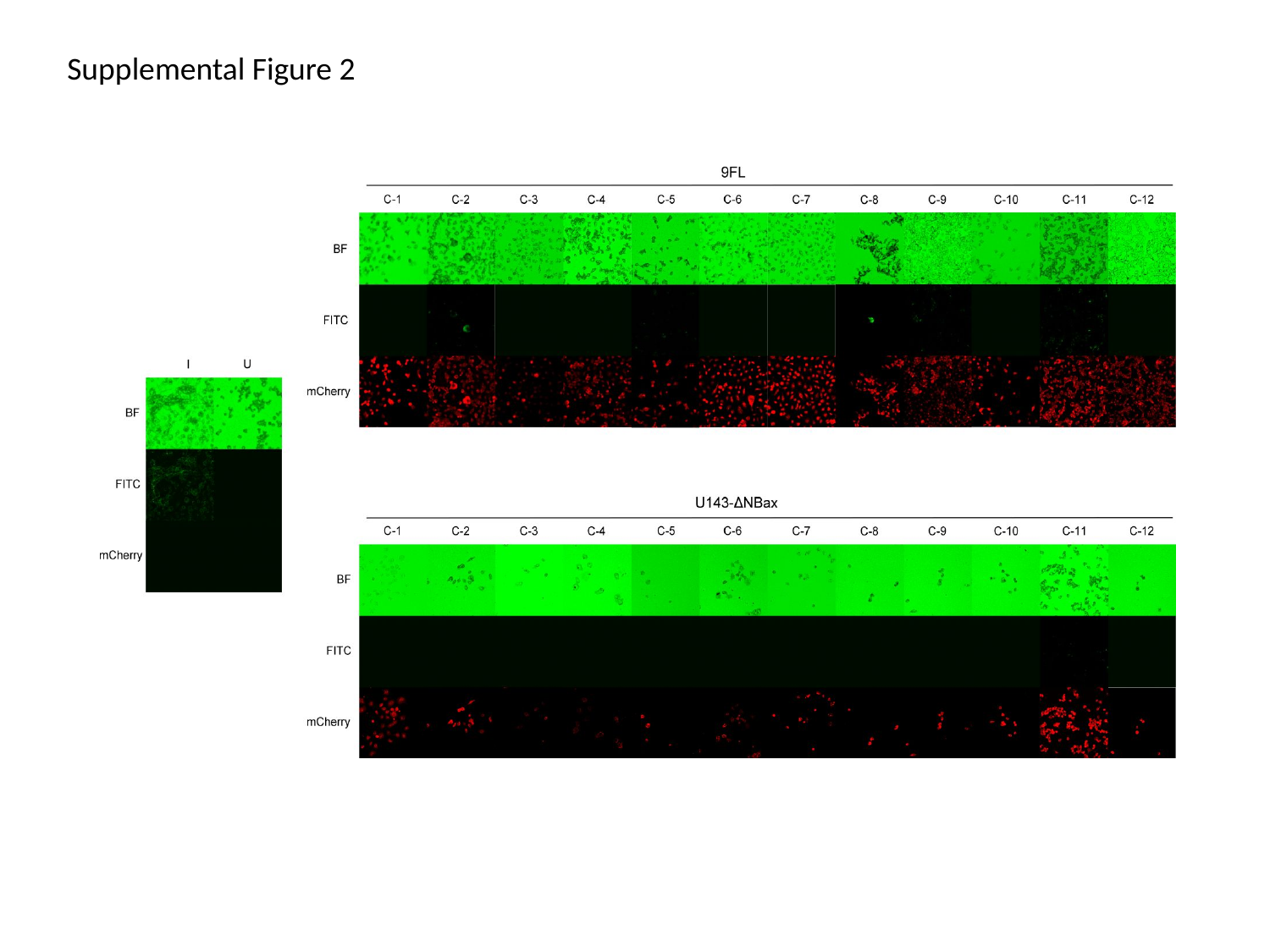

Supplemental Figure 2

Supplement: Additional file 2: Figure S2 — Clonal cell populations (labeled C-1 through C-11) were challenged with DENV-2 NGC (MOI = 0.01). At 4 dpi cell supernatants were collected and saved for RT-PCR analysis (see 8b). Following DENV-2 E protein antigen staining with antibody, micrographs were taken using the A1-R confocal microscope (Nikon). I = infected, U = uninfected. [file 1743-422X-11-111-S2.pptx]

## Slide 1
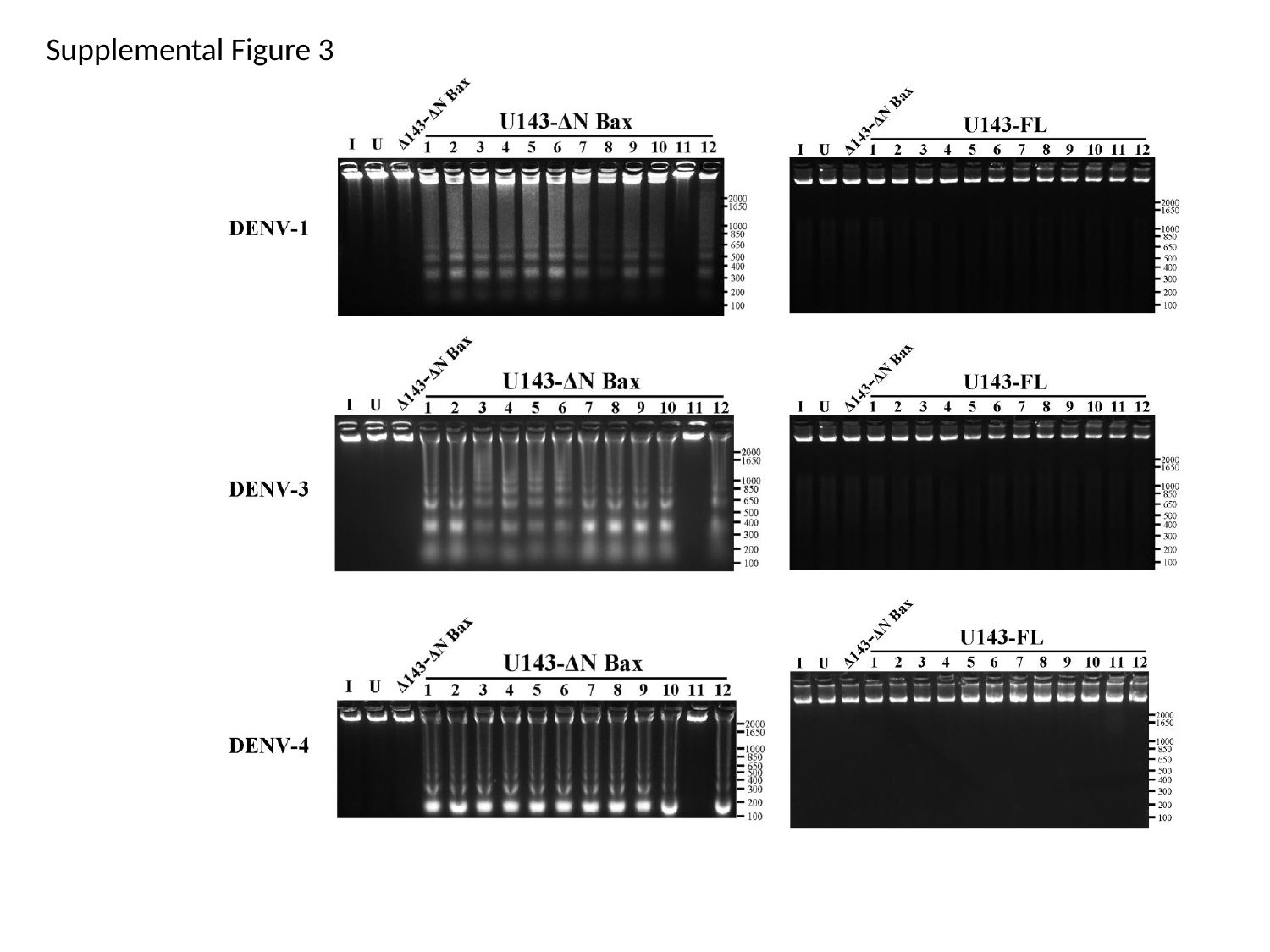

Supplemental Figure 3

Supplement: Additional file 3: Figure S3 — Clonal cell populations stably expressing αDENV -U143-FL, αDENV -ΔU143-ΔN Bax or αDENV-U143-ΔN Bax (labeled C-1 through C-11) were challenged with DENV-1, DENV-2 (see Figure 9) DENV-3, or DENV-4 (MOI = 0.1). At 4dpi analysis of DNA fragmentation was performed as described in Methods. I = infected, U = uninfected. [file 1743-422X-11-111-S3.pptx]

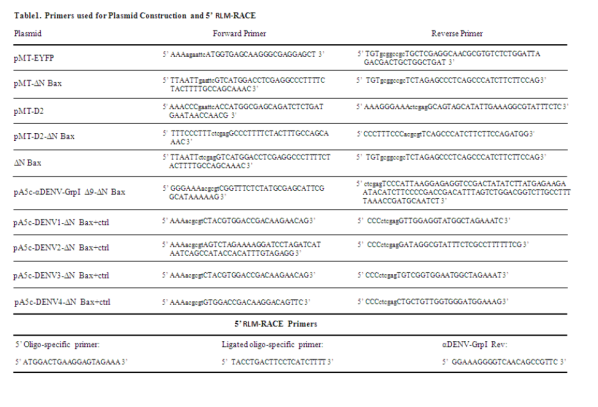

Supplement: Additional file 4: Table S1 — Primers used for plasmid construction and 5’ RLM-RACE. Listed are the forward and reverse primer sets used to produce the PCR fragments and 5’RLM-RACE analysis. Lowercase nucleic acids indicate restriction site. See Methods for description of vector constructs. [file 1743-422X-11-111-S4.doc]
